# Recent Advancements in Digital Management and Monitoring of Mine Waste: Sensors, Characterization, and Predictive Modeling—A Review

**DOI:** 10.3390/s26113553

**Published:** 2026-06-03

**Authors:** Tianqi Li, Feven Desta, Mike Buxton

**Affiliations:** Geoscience and Engineering Department, Delft University of Technology, 2628 CN Delft, The Netherlands; f.s.desta@tudelft.nl (F.D.); m.w.n.buxton@tudelft.nl (M.B.)

**Keywords:** mine waste monitoring, sensors, characterization, modeling, artificial intelligence

## Abstract

Mining activities generate substantial volumes of solid waste materials during exploration and processing. These residuals pose environmental and geotechnical concerns due to their large spatial footprints and associated risks but may also contain potentially valuable resources. These characteristics highlight the necessity and opportunity of effective management and monitoring strategies. In recent years, a diverse range of technologies and methods have been applied to characterize mine waste compositions and analyze their spatial–temporal variability. These include remote sensing systems, ground-based sensors, and advanced data-driven methods. Despite the rapid advancement, the existing literature provides limited insight into the critical evaluation of how these techniques are applied in practice. This review systematically examines peer-reviewed journal articles published between 2021 and 2024 to highlight the state of the art in characterization, modeling, and monitoring techniques for mine waste. The review identifies recent trends, key gaps, advantages, and limitations of these techniques. The summary suggests that mining companies and research communities are increasingly adopting innovative technologies, transitioning from conventional methods to more sustainable practices. However, it also reveals ongoing challenges and persistent limitations. Further efforts, such as real-time monitoring capabilities, are required to achieve full implementation and integration across the industry and academia.

## 1. Introduction

Mining is one of the most crucial sectors in global industry. It not only drives significant economic growth worldwide and supplies essential mineral resources for diverse industries but also provides substantial employment opportunities. Mineral resources are vital for global energy demands and supporting the transition towards cleaner production. However, the exploration, extraction and processing of mineral deposits generate large volumes of inevitable waste materials. Valuable materials in the ore only exist in low proportions. In the case of some metals, such as Au, the ratio of the valuable extraction to waste generation can reach as high as 1:12, meaning that for every ton of metal mined, up to 12 tons of waste is produced [1]. This ratio depends on several factors, including ore type, the mining method beneficiation process, and the cut-off grade. For example, lower-grade ores require processing larger volumes of material to extract the same amount of metal compared to high-grade ores. As estimated, 183.35 billion tons of waste was accumulated from mining activities in 2023 worldwide [2,3]. It has been identified as the major waste stream in many countries.

Mining waste can exist in various forms, including solid, liquid, gaseous, and mixed phases [4]. Among them, solid mining waste is the most prevalent and is generated throughout the entire mining value chain. These waste streams include waste rock, tailings, and slags. Waste rock consists of rocks and soil removed to access the ore during the early extraction stage. Tailings are the finely ground residues left after ore processing and milling. Slags are the by-product of the metallurgical smelting and refining [5]. They often occupy large areas of the natural land and can pose serious environmental and safety risks if not properly managed and monitored.

One of the most pressing concerns related to mine waste is the dispersion of potentially toxic elements (PTEs) from the waste piles to the nearby environmental system [6]. This can occur through several processes, for example, weathering, erosion, and run-off. These activities transport the residual heavy metals into the surrounding soil, water, and vegetation, which further affects the health of the local community through these environmental media [7,8]. This issue is especially pronounced for historical waste sites, which are often closer to residential areas and were developed before strict regulations were in place [9].

Tailings are typically stored in various forms, such as slurry deposits. Tailings storage facilities (TSFs) are engineered structures designed for the centralized, long-term management of these materials. As tailings accumulate within TSFs, the increasing volumes and dynamic weather can make them prone to failure, causing landslides, dam breaches, and other hazards. These events not only lead to severe contamination of the downstream ecosystem through the direct release of toxic elements but also endanger human lives. In a recent TSF failure incident in Brazil, the collapsed slurry traveled at up to 120 km/h, causing 267 fatalities, and several people are still missing [10].

Despite these challenges, mine waste may also contain significant untapped resources. This can happen due to a range of factors, such as cut-off grade change, historical processing inefficiencies, and the shift of interests in minerals over time [5]. Technological advancements can make uneconomic materials become attractive. Therefore, many mine wastes still host considerable concentrations of metals and minerals. Since the depletion of higher-grade ores and to ensure autonomy in mineral supply, these secondary resources are emerging as alternatives to satisfy the increasing demand for minerals driven by population growth, green energy transition, and emerging technologies [11].

Effective and proactive mine waste management, together with long-term monitoring, is crucial for reducing the potential environmental impacts and safety risks associated with these wastes, while also maximizing the resource potential for sustainable mining. Achieving this goal requires a thorough characterization of the physical, mineralogical, and chemical composition of mine waste, as well as modeling its dynamics across spatial–temporal scales. Many key parameters are highly associated with the potential risks of mine waste. For example, the surface pH at waste sites could indicate the potential of acid mine drainage (AMD) formation and chemical stability [12]. In addition, at many former uranium sites, the radioactive elements can migrate to the surrounding ecosystem and harm the nearby communities. Identifying such variables not only enables early detection of environmental contamination but also informs the development of predictive models and long-term remediation strategies.

The management and monitoring of mine waste face several challenges. Each mine waste site is unique. The physical, chemical, and mineralogical characteristics of these residuals can be highly site-specific due to the geological setting, original commodities, extraction methods, site climate conditions, and the microbial community formed within the piles [13]. In some cases, several open-pit projects may share the same TSF, with waste materials deposited over different stages of operation. As a result, the overall composition of mine waste is often highly complex, reflecting the heterogeneous and dynamic nature of the deposited materials. Moreover, their volumetric characteristics are often unknown due to measurement difficulties, adding further uncertainty to their management and assessment.

Beyond these characteristics, traditional techniques for managing and monitoring these materials also have notable limitations. These include high operational costs, limited resolution and accuracy, low level of automation, and potential safety risks. For example, laboratory-based approaches require operation in controlled environments and cannot easily be deployed in the field. Mathematical methods often provide only simple calculations and produce rough, less accurate results. Point-based sampling may not capture the high-level heterogeneity within the residuals. The available data remains limited due to the feasibility of field campaigns for sample collection. Due to confidentiality issues, many datasets are also not readily shareable, which further constrains the research.

To overcome these limitations, researchers have increasingly adopted innovative techniques and approaches. With the ongoing advances in technologies and concepts, various modern equipment, methods, and algorithms are now being applied to the management and monitoring of mine waste. Recent developments in sensor-based technologies and data-driven methods have significantly enhanced the ability to characterize, model, detect, predict, and monitor these waste materials. Ground-based sensors could provide accurate characterizations of mine waste samples; remote sensing platforms, with their high spatial, temporal, and spectral resolution, have expanded opportunities for monitoring mine waste across a range of scales; geospatial methods help overcome the limitations of traditional field approaches; AI techniques support the automation of complex data processing. These technologies can be used for a range of applications, from material characterization to advanced prediction and novel monitoring systems in different fields such as geoscience and the environment.

The environmental issues and resource potential of mine waste are closely connected to the broader ecosystem, influencing land, water, biodiversity, and community. Therefore, understanding the current technological advancement is crucial for effective mine waste management and monitoring. In recent years, several review articles have addressed the technical aspects of different phases of mining activities. Responding to the growing social concerns on geohazards, Cacciuttolo et al. [14] reviewed the application of sensor technologies for monitoring the safety of TSFs, covering tools such as remote sensing, aerial sensors, and the Internet of Things (IoT). They highlighted the gap of effective communication platforms that provide reliable real-time information to communities and identified several key parameters that require attention under climate impacts. Similarly, Choi et al. [15] systematically reviewed the application of a geographical information system (GIS) across three mining stages—planning, operation, and environmental management—emphasizing the GIS as a powerful and flexible tool for mining management and decision making. More recently, a study published in 2025 systematically reviewed mine tailings research over the past thirty-four years, with a particular focus on geostatistical models, recovery and revalorization approaches, and the economic potential of mine waste materials [16].

These reviews demonstrated the growing role of advanced technologies in addressing challenges associated with mining activities and provide valuable insights into the research trend. However, they primarily focus on specific techniques, broader mining operational life cycle, or long-term publication patterns. Recent reviews tend to emphasize trend analysis on bibliometric information rather than in-depth discussion across multiple technologies. Building on these efforts, there remains a lack of comprehensive synthesis that reviews multiple advanced techniques, especially both sensor-based and data-driven approaches, for the characterization and modeling of mine waste.

To address this gap, the present review aims to provide an updated overview of recent advancements in the management and monitoring of mine waste, with a primary focus on the latest trends over the past four years, from 2021 to 2024. It captures the recent innovations, identifies critical gaps, and highlights emerging sensor technologies and data-driven methods. Earlier studies and the most recent work from 2025 are incorporated for comparison, in order to identify shifts in research priorities and reflect the evolving landscape of management and monitoring approaches.

The scope of this review is defined to ensure clarity in topic coverage and boundaries. It focuses on solid mine wastes, for example, waste rocks, tailings, and slags. This review examines journal articles that apply advanced sensor-based technologies and data-driven methods for characterization, modeling, management and monitoring purposes. Other physical entities, such as liquid and gaseous mine waste, water, and soil are outside the scope. Studies focusing on the chemical or physical extractions and direct material recovery are also excluded. The systematic analysis highlights journal articles published between 2021 and 2024, with earlier work and recent studies from 2025 incorporated selectively to provide background context. Detailed inclusion criteria are provided in Section 2.2.

It is worth noting that this review is not intended to focus on the detailed technical aspects of these included methods and algorithms. This review systematically summarizes the selected articles, presenting an overview of the current state of the art, highlighting the current challenges and limitations, identifying the research gaps, and proposing future research directions.

This article is structured as follows: Section 1: introduction of background and this review article; Section 2: methodologies used for collecting review articles; Section 3: results from systematic review of each technology and method, described in text, graphics, and tables; Section 4: discussion of opportunities, challenges, limitations, and environmental and social considerations; Section 5: the conclusion summarizes the key findings and proposes directions for future research.

## 2. Methods

**Figure 1:**
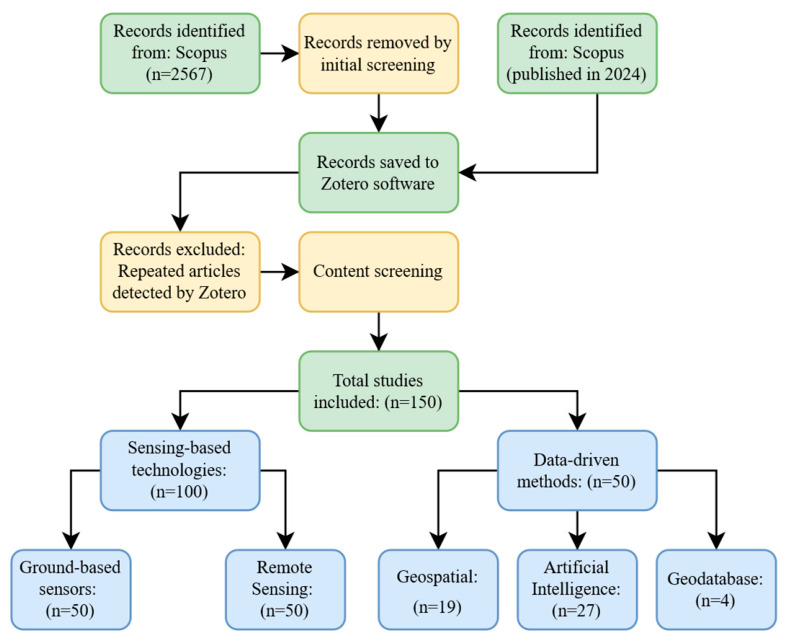
Workflow illustrating the collection, screening, and categorization of articles included in the review.

This section outlines the methods used to conduct this review. The collection, selection, and analysis of the articles have referenced the Preferred Reporting Items for Systematic Reviews and Meta-Analyses (PRISMA) 2020 statement for systematic reviews [17]. A workflow (Figure 1) was generated using draw.io to illustrate the literature collection process and the number and division of the selected papers. A bibliometric analysis of the keywords from the collected articles was conducted to obtain an initial understanding of the research trend. The co-occurrence maps of the keywords were generated separately for different collections of techniques using the VOSviewer software 1.6.20. RStudio 2024.04.0 is used to generate statistical graphics used in Section 3.

### 2.1. Literature Collection

The collection of the literature was performed using Elsevier’s Scopus search engine. While Scopus generally includes a broader spectrum of journals, it also surpasses Web of Science specifically in the field of natural science and engineering, particularly in its unique articles [18], which align better with the field of mining waste studies. A structured literature search was conducted between September and October 2024 to establish an initial understanding of the current techniques. A follow-up search was carried out in March 2025 to capture relevant publications from late 2024 that were not available in the initial search. While the core systematic review focuses on articles published between these four years, earlier and recent 2025 studies were also searched using the same search strategy. Representative articles from these time frames are selectively incorporated in the discussion to support the analysis.

Several keyword combinations using Boolean operators were used as search strategies to cover multi-disciplinary studies. The study objects were described as (“mine waste” OR “tailings”). The keywords for the study objects and technologies were connected with “AND”, while “OR” was used among the technologies. Research articles published in international journals after peer review were collected. To ensure an accurate understanding of the content, the language filter was set to select articles in English only. Based on the category of technologies and methods, keywords were divided into seven groups and searched separately. This is to reduce the potential of Scopus crashing. In total, 2567 search results were initially obtained, including overlapping records.

After completing each search group, the titles were screened to identify relevant papers using several pre-defined selection criteria. Studies that could not be distinguished by title, abstract, keywords, and conclusion were screened for further decision. The selection process followed a rigorous methodology to minimize bias and provide a comprehensive synthesis of current advancements. Representative papers were imported into Zotero 7, a citation management software. Zotero automatically detects duplicate articles in the “Duplicated Items” folder and then removes them manually. During the review, an Excel spreadsheet was used to record the metadata of the papers, the main research objectives, methodologies, and findings for further comparison and analysis.

### 2.2. Selection Criteria

Various research methods and analytical techniques have been used to yield valuable insights. However, strict selection criteria were necessary to filter the most relevant and reliable studies. This is not only to ensure the credibility of the included research but also to enhance the quality of the discussion and maintain a focused scope that is aligned with the objectives of this review.

The criteria used to decide whether an article would be fully reviewed and statistically summarized were defined as follows:

1. Focused on solid mining wastes, for example, waste rocks from extraction, tailings from processing, and slags from metallurgy. Liquid and gas waste, or research focused on water bodies and soil, are outside the scope. Some research focused on a wide range of media and, in this case, they are excluded if mining waste is not the focus.
2. The research methods include the utilization of advanced sensor-based and data-driven techniques for mining waste. Some research implemented these methods but aimed only to validate results from other techniques, or did not reveal any insight into the waste entities, and was excluded. Studies that rely solely on traditional chemical methods, such as laboratory leaching experiments, and on basic statistical calculations, such as simple linear regression, are excluded from the systematic analysis. Still, representative articles will be included in the discussion.
3. The research purposes are related to the revalorization, management, or monitoring of mining waste, particularly from the perspectives of sustainable development. Studies that focused solely on physical and chemical methods for direct material recovery from tailings were excluded.
4. To capture the recent advancements, a time frame was set for this review that only journal articles published online between 2021 and 2024 will be included in the systematic analysis. Articles outside this time frame are incorporated selectively to provide background and to keep up with progress.

### 2.3. Classification of Sensors and Analytical Techniques

Following the selection process, the selected studies were classified according to the primary technologies and analytical approaches used. This review encompasses two main categories of techniques for managing and monitoring mine waste: sensor-based technologies and data-driven methods.

Sensor-based technologies were further grouped into two categories: ground-based sensors and remote sensing technologies. In this review, remote sensing refers to the techniques that acquire data from above Earth’s surface at a distance. To enhance clarity, satellite-based, airborne, and light detection and ranging (LiDAR) devices are grouped under the category of remote sensing. Airborne and satellite platforms collect data from aerial and orbital positions, and LiDAR could be mounted on either platform to perform measurements. Although LiDAR can also be operated from ground-based platforms, only LiDAR systems mounted on airborne or satellite platforms are considered as remote sensing in this study.

It is acknowledged that some ground-based sensors, such as ASD field spectroradiometers and close-range hyperspectral imagers, can also operate remotely without direct physical contact. They align with the definition of proximal remote sensing. However, they are included in the ground-based category in this review due to their relatively fixed, close-range operations, their limited distance from the target surface, and their small-area measurements. These factors, along with the deployment platforms, distinguish them from large-scale remote sensing applications.

Data-driven methods in this review comprise analytical domains (geospatial and AI-based methods) and supporting data infrastructure (geodatabase). Geospatial methods primarily refer to techniques that consider spatial features, including mapping analysis, geostatistics, interpolation, temporal modeling and scenario visualization. AI-based methods primarily refer to ML approaches for pattern recognition, automation, classification, segmentation, and prediction, which may not explicitly consider spatial structure. This classification is intended for a clear organizational framework and does not represent mutually exclusive categories. In practice, overlaps exist between geospatial and AI-based methods such as creating 2D surfaces based on point measurements. Machine learning (ML) is increasingly being incorporated into spatial information process workflows. Geodatabases are considered the foundation of data-driven methods, as they support the storage, organization, and management of spatial mining waste data for further analysis.

In conclusion, the included articles were grouped as follows: ground-based sensors, remote sensing technologies (satellite, airborne, and LiDAR), geospatial methods (including geostatistics and spatial modeling), geodatabases, and AI-based methods.

### 2.4. Co-Occurrence Mapping

To initially identify the research “hotspot” and visualize thematic trends, keyword co-occurrence network maps were generated separately for ground-based sensor studies, remote sensing studies, geospatial studies, and AI-based studies. Geodatabase studies were excluded from this analysis as only 4 publications were identified that met the selection criteria and time frame. The amount is insufficient to produce a meaningful co-occurrence network.

Bibliographic metadata were exported from Zotero in RIS format and processed using VOSviewer. The keywords were selected as the unit of analysis, and a minimum occurrence threshold of 1 was applied, given that these are relatively small collections. In the co-occurrence maps, larger node sizes indicate higher keyword occurrence frequencies, and link thickness indicates the strength of co-occurrence between keywords. The sequential overlay color scale reflects the publication year of the keywords, with darker colors indicating more recent research topics. Different color themes were applied to individual maps to improve visual differences among study categories.

As VOSviewer exports static screenshots of the networks with partial label suppression to reduce overlap, visualization parameters, such as scale, node size variation, orientation, and color settings were adjusted to improve readability across the figures.

## 3. Findings and Analysis

### 3.1. Overview of the Screening Result

In total, 2567 results were obtained from the search. After removing duplicates and applying the exclusion criteria described in Section 2.2, a total of 150 papers were identified and included in this review. The included studies were assigned to two primary domains based on the defined scopes: 100 articles were identified for sensor-based technologies (including ground-based sensors and remote sensing), and 50 articles were included for data-driven methods (including geospatial methods, geodatabase, and AI). Figure 1 shows both the detailed screening and selection process and the overall distribution of articles among the categories.

### 3.2. Sensor-Based Technologies

Sensor technologies are crucial in mine waste management and monitoring. These technologies can be classified into two major categories: ground-based sensors, including lab-based sensors and portable field sensors, which may operate through either direct or proximal measurement, together offering highly accurate localized information; and remote sensing technologies, including satellite and airborne sensors, which enable large-scale and flexible spatial–temporal assessment. Recent advancements in both categories have enhanced their precision, integration, and have demonstrated their capabilities across various applications.

This section presents the findings on ground-based sensors and remote sensing, focusing on the sensor types, applications, and key highlights. As in most papers that apply ground-based sensors, these paper focus on site-scale characterization, while remote sensing studies use ground-based sensors mostly only for validation. These two parts will therefore be presented separately in Section 3.2.1 and Section 3.3.2 and discussed together in Section 4.1.

*Co-occurrence of Keywords and Emerging Theme*

**Figure 2:**
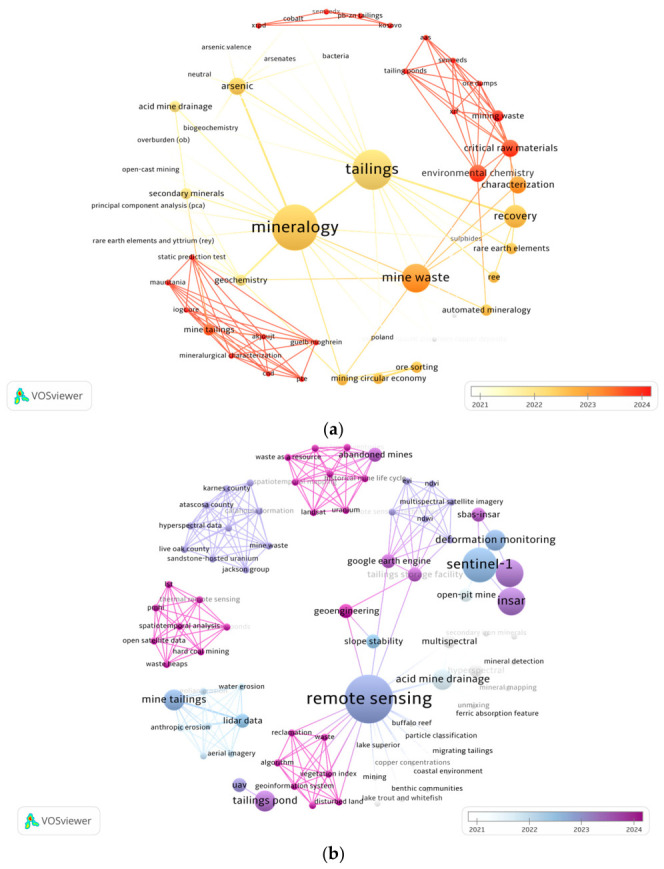
Keyword co-occurrence analysis of reviewed studies on sensor-based technologies for mine waste monitoring and management: (**a**) Ground-based sensors articles; (**b**) Remote sensing articles.

The co-occurrence analysis revealed several distinct characteristics within each technology group. In the ground-based sensor collection (Figure 2a), the major themes include “tailings”, “mineralogy”, “recovery” and “arsenic”. Recent studies increasingly emphasize the terms “characterization” and “critical raw materials” (CRMs). In the remote sensing collection (Figure 2b), a distinct and relatively recent theme could be identified, which includes the high-frequency terms “Sentinel-1”, “interferometric synthetic aperture radar” (InSAR), “deformation monitoring”, and “tailings dams”.

#### 3.2.1. Ground-Based Sensors

Ground-based sensors have been widely applied to provide multi-dimensional insights into raw material characterization, including mine waste. These sensors operate based on different principles and are designed to detect various aspects of material properties. They encompass a variety of analytical techniques and could enable rapid and accurate assessment of the mineralogical, chemical, and physical properties of residuals. Such characterization is an essential step in mine waste management and monitoring. It provides key data to support applications, including environmental assessment, identification of potential resources, and valorization of waste materials.

The effectiveness of these sensors for material characterization depends on several factors, including their operating principles, resolutions, calibration, field environmental conditions, and the effectiveness of the selected data processing methods. Table 1 provides common application examples for the most frequently used sensors.

A total of 50 articles were included in the ground-based sensor category. The mentioned purposes vary among:

- Environmental monitoring (*n* = 15): to assess AMD (e.g., [19,20]), PTE (e.g., [21,22]).
- Resource identification (*n* = 14): for example, evaluating the potential of precious metals [23], REE recovery [24], and other CRMs [25].
- Valorization (*n* = 11): to upcycle the waste materials (e.g., [26]).
- Some studies focused on multiple purposes, as environmental problems can sometimes be considered a potential resource when treated properly (*n* = 7; e.g., [27]).
- Methodological innovation: three other articles focused on improving methodology for understanding uncertainty [28] and for data fusion [29,30].

Table 2 provides an overview of the studies included, summarizing their main research purposes.

**Figure 3:**
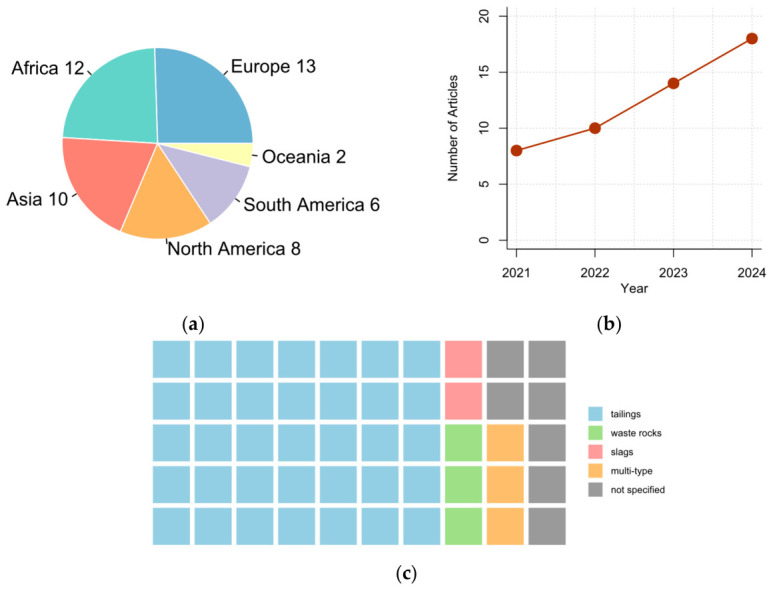
Characteristics of the reviewed ground-based sensor studies, including geographical, temporal, and waste-type distribution: (**a**) Geographical distribution of the studied sites. Note: one article has focused on sites across two continents; (**b**) Temporal distribution of the articles over the past four years; (**c**) Waste-type distribution.

As illustrated in Figure 3a, the selected articles cover various waste sites across six continents: the waste sites studied in Europe (*n* = 13), Africa (*n* = 12), and Oceania (*n* = 2) mainly aim at resource recovery potential; North America (*n* = 8) primarily investigated environmental monitoring; Asia (*n* = 10) and South America (*n* = 6) present mixed purposes. One article has investigated sites in both Asia and Europe. The geographical distribution of research focus reflects regional differences in mining activities and economic contexts. For example, despite Europe being the only continent to show a decrease in mining activity in recent years, demand for raw materials remains high due to the energy transition, which may explain the stronger emphasis on resource recovery. In contrast, countries in North America, which are major producers of minerals rather than importers, may prioritize mitigating environmental impacts over resource recovery.

Beyond the spatial distribution, Figure 3b illustrates the temporal trends in the number of published articles within the defined time frame. The short-period trend reflects the most recent dynamics. Additionally, a temporal shift in interests among articles was observed: in 2022, resource identification dominated; in 2023, shifting to environmental monitoring; and in 2024, renewed interest in resource recovery emerged, while valorization was also discussed more frequently. The increased interest in these resources reflects growing concerns about raw material availability and supply chain stability. This trend may have been influenced by geopolitical uncertainties, which highlighted the need for diverse material sources, and increased countries’ autonomy in the global market. The shift in interest toward environmental monitoring may be driven by the growing emphasis on responsible, sustainable mining practices, supported by green energy policies. This also explains the renewed interest in CRMs such as REEs, which are essential for the clean energy transition. This observation aligns with the initial findings from the keyword co-occurrence map.

From a material perspective, Figure 3c shows the distribution of waste types being analyzed across the collected articles. The majority (70%) focused on tailings, 6% on waste rocks, 4% on slags, and 6% on multiple waste types. This indicates a strong emphasis on tailings, likely due to their large volume and surface occupation, dynamic nature, complex reaction with different environmental systems, and potential for both contamination and resource recovery.

The reliability and representativeness of the reported findings may be influenced by several sources of bias and uncertainty, primarily related to sampling strategies and reporting transparency. Multiple studies relied on relatively small sample sizes (<15 samples) [29,34,44], which may not adequately capture the spatial and compositional variability of the waste site. Small datasets in mine waste site characterization increase uncertainty, reduce the ability to capture spatial and geochemical variability, and limit the effectiveness of statistical and predictive models. This can lead to inaccurate revalorization potential assessments, environmental risk assessments and less effective remediation decisions. For example, mine waste sites are highly heterogeneous, with waste rock piles, tailings, drainage pathways, mineral composition, and contaminant concentrations often varying significantly over short distances. Small datasets may overlook key spatial patterns or localized contamination “hotspots”, resulting in site characterizations that do not accurately reflect actual conditions. Furthermore, incomplete reporting of sampling procedures, calibration settings, and analytical workflows across several studies limits the systematic comparison among studies. Variations in sampling method, sampling density, and pre-processing methods for raw sensor data may explain inconsistencies in reported findings.

The combination of sensors has demonstrated promising effectiveness in understanding waste materials. In mineralogical analysis, XRD is the most commonly employed analytical technique, which is often combined with SEM-EDS. While XRD generates diffraction patterns that reveal the crystallographic structure and phase composition of a material, SEM-EDS provides high-resolution visualization together with microstructural and elemental mapping. Their integration improves the confidence and accuracy in mineral identification. For elemental characterization, XRF is the most popular sensor, with ICP-MS for validation. XRF delivers elemental profiles, whereas ICP-MS provides substantially higher analytical sensitivity for trace elements below XRF’s detection limits. These combinations generally improve the analytical reliability.

Toxic elements, heavy metals, and AMD-related elements are the primary focus due to their persistence, accumulation and harmful effects on the ecosystem and surrounding communities. Among the reviewed articles, Fe, S, and As appear to be the most problematic elements [20,22,25]. Through oxidation, weathering, and other processes, waste materials containing these elements can pose a significant environmental risk. They may generate AMD, particularly at historical sites that have been exposed for long periods [26]. Arsenic occurs in diverse and complex combinations within waste materials. SEM-EDS observation indicates associations between As, Fe oxides, and arsenopyrite, influencing contaminant mobility [30]. At another site, the combination of sensors and leaching tests confirmed that waste rocks have the highest As leaching and the least stable geochemical conditions compared to tailings and ores from the same location [25]. These characteristics directly affect remediation difficulties. In the Long Lake gold mine region, sulfide mineral oxidation products were transported upward through the historically remediated tailings and contaminated the sand cover placed for environmental protection [22]. The shallowing pore water was found to contain high levels of As, Fe, and SO_4_ [22]. The remediation therefore became secondary pollution. In addition to the elements mentioned above, residuals from the original commodities may contain other harmful elements. For example, gold mining can result in high levels of Hg (50 ppm) [39], while Cu, Co, Ni, Sb, and Se may contribute to the formation of contaminated neutral mine drainage [23]. Residuals may also contain Pb [33] and Cd [36]. In phosphate tailings, radioactive elements were also found to be exceed the safety standards recommended by the International Atomic Energy Agency [29]. These findings suggest that environmental risks vary according to waste type, mineralogy, and extraction methods and therefore require site-specific characterization.

Resource potential assessment reveals base, precious, and critical resources at many studied sites, occurring in tailings, waste rock, overburden, and slag. The economic value and ease of recovery vary across different waste sites. In some places, tailings contain base metals of Cu, Pb, and Zn with certain economic interests (2500 ppm; 2500 ppm; 1500 ppm) [48]. Some waste piles contain high concentrations of REEs, from an average of 26.3 ppm up to 5650 ppm [34,43]. The concentration could exceed some low-grade primary ores [44]. In some cases, the tailings contain precious metals of commercial value [39]. As one of the major topics in secondary resources, critical elements were also found within many waste sites. For example, high levels of Bi (35,490 ppm) and Sb (15,930 ppm) were found at sites in Romania [52]. Some sites show great economic potential [36], but some reported difficulties in reprocessing due to the heterogeneity of the waste piles [38]. This highlights the need for a comprehensive analysis that incorporates both mineralogical and geochemical information.

Data fusion has been an emerging research method and has shown improved performance in many cases compared to single sensors, especially for Fe-related content. When fusing the VNIR and TIR by the outer product analysis (OPA) method, the random forest prediction accuracy in R^2^ of TFe and SiO_2_ content improved from 0.70 to 0.91 and from 0.67 to 0.95, while the RMSE decreased from 1.60% to 0.96% and from 2.49% to 0.97%, respectively [63]. This same trend of accuracy is also shown by Kamps et al. [64]. Although improvement was not the case for all analyzed elements, the prediction performs best in full-range infrared for Fe content and VNIR fusing with short-wave infrared (SWIR) for Ti. For other elements, like Sr and Rb, FTIR at a longer infrared range performs better with R^2^ > 0.7.

The reported findings showed variabilities in environmental risks and resource potential. Such differences are partly associated with site-specific characteristics, including geological setting, ore type, extraction planning, metallurgical efficiency and processing history, which influence the waste composition and total volume of materials. Together with local mineralogy and geochemistry, these factors determine the complexity of treatment and recovery. For example, in environmental monitoring studies, while some residuals are identified as a “high risk” to the environment due to heavy metal leaching, some waste materials are considered “harmless” [32]. This variation is influenced not only by the inherent characteristics of the materials but also by the efficiency of the metallurgical techniques used during the ore processing. Some techniques minimize residual contaminants, while others, such as whole ore amalgamation (WOA), require large amounts of harmful elements as treatment methods and capture only 30% of the precious commodity [39].

Beyond site-specific factors, variability in findings also arises from methodological inconsistencies, including the deployed sensors and sampling strategies, and the raw output processing method, which complicates the systematic comparison across studies. Some studies used systematic sampling methods (e.g., [37]), while others relied on random sampling strategies (e.g., [56]), potentially affecting representativeness and uncertainty.

Additionally, the selection and combination of sensors further contribute to variation, because different sensors are sensitive to different material properties and mineralogical conditions. Each sensor has its own detection limitations. Some sensors demonstrate high accuracy for specific elements or mineral phases, and their performance may decline or be inapplicable to other waste with different geological settings and elemental compositions. Surface materials exhibit distinct reflectance signatures across the electromagnetic spectrum, and certain wavelength regions are more suitable for detecting particular materials. For example, Fe-bearing minerals commonly show diagnostic signals in the VNIR range. In contrast, silicate features are often more detectable in the long-wave infrared region, consistent with FTIR’s detection capabilities. As a result, sensors that perform well for one type of waste may have limited applicability in regions where materials lack distinct signals within the sensor’s detection range. This may partly explain inconsistencies in reported performance across studies and highlights the importance of selecting sensors according to the specific characteristics and type of waste material, rather than assuming universal applicability.

In general, a wide range of ground-based sensors have demonstrated rapid, accurate characterization capabilities for understanding the mineralogical, chemical, and physical properties of mine waste, providing essential information for environmental assessment and resource recovery. Multi-sensor combinations and data fusion approaches generally provide more reliability and comprehensive characterization results than individual sensors. However, their effectiveness is still influenced by factors such as sampling representativeness, local geological variabilities, and sensor capability for materials. Future studies would benefit from more standardized sampling reporting documentation.

#### 3.2.2. Satellite- and Airborne-Based Remote Sensing

Remote sensing technologies have emerged as powerful tools for monitoring and assessment of mine waste, enabling large-scale, continuous spatial and temporal analysis. The main categories covered by this review are the use of satellite- and airborne-based remote sensing techniques. These platforms can accommodate a wide range of sensor equipment. LiDAR, an active sensing technique deployable from ground, aerial, or mobile platforms, has primarily been applied to high-resolution terrain reconstruction and volumetric assessment.

A total of 50 studies utilizing remote sensing were identified and included based on the selection criteria and their representativeness. They are grouped into satellite (*n* = 32), airborne (*n* = 14), and LiDAR (*n* = 4). A few studies are highly relevant but were excluded due to accessibility and differences in research targets [66]. To enhance clarity, Table 3 summarizes the satellite-based articles, while those using airborne and LiDAR are summarized in Table 4 and Table 5, respectively. Notations of application purpose, measurement parameters, and studied locations are included in the table.

**Figure 4:**
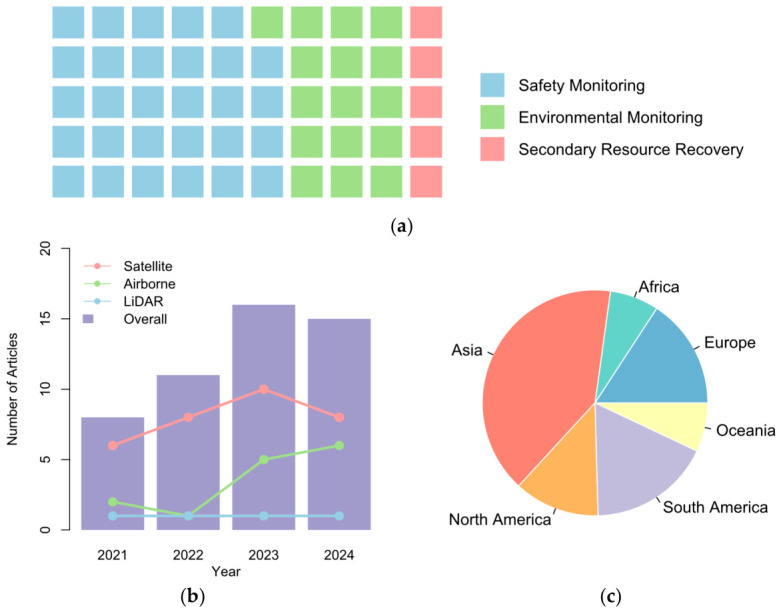
Overview of research trends and characteristics in satellite- and airborne-based remote sensing studies: (**a**) Research purposes distribution; (**b**) Number of the reviewed articles by year; (**c**) Geographical distribution of studied sites based on the reviewed articles.

The application purposes of remote sensing technologies could mainly be divided into monitoring, detection, mapping, and characterization of mine waste materials. These application purposes are linked to the research purpose (Figure 4a) in safety monitoring (*n* = 29), environmental monitoring (*n* = 16), and secondary resource recovery (*n* = 5). Among the safety monitoring applications, two common ones are identified. The first theme is stability monitoring, particularly the assessment of tailings dam failure risks, slope stability, and deformation. Another theme is the spontaneous combustion hazard, specifically for coal mine waste. Apart from the dominance of satellites across the three research purposes, LiDAR devices stand out in environmental monitoring (*n* = 4), particularly for geomorphological assessment. This distribution suggests that platform selection is strongly influenced by monitoring objectives.

In addition to the application focus, the temporal distribution of the included studies reveals an overall increase in interest in remote sensing for mine waste monitoring in recent years. Based on the reviewed articles within the specified time frame, the number of publications peaks in 2023, with 16 publications, as shown in Figure 4b. This trend is mainly driven by the use of satellites, which aligns with the overall pattern. In parallel, studies using airborne platforms have shown a steady increase, reflecting advances in airborne technology and a better understanding of their usage regulations.

The geographical distribution of the studied locations spanned six continents, indicating global interest in the exploration of remote sensing for mine waste applications (Figure 4c). Based on the reviewed articles, the most frequently studied sites are in Asia, with 23 articles mentioning them, with more than half (*n* = 13) in China. Study sites in South America (*n* = 10) are mainly reported in satellite-based articles (*n* = 9), primarily in Chile and Brazil. One of the possible reasons is the significant tailings dam failure, the Brumadinho incident, which happened in Brazil a few years ago, which has been a reference in many methodological articles that trace back to failure signals. Study sites from Europe are mentioned in nine articles, with a balanced use of three platforms and devices. Study sites in North America (*n* = 7) exhibit a similar trend, potentially indicating greater accessibility of diverse data sources and devices. Sites in Oceania (*n* = 4) and Africa (*n* = 4) share similar trends, which are heavily based on satellite studies. It should also be noted that many articles have focused on several study sites across more than one continent, which highlights the advantage of remote sensing in enabling data acquisition where field access is difficult, hazardous, or economically constrained.

The satellite sensors used across the articles vary in spatial, temporal, and spectral resolutions, each offering unique advantages across periods and regions. A summary of the parameters of the mentioned sensors across satellite-based studies is shown in Table 6. The most widely used satellites include Sentinel-1, Sentinel-2, and Landsat 8, offering open-access, consistent data. Sentinel-2 and Landsat 8 are commonly used for environmental monitoring and for assessing the potential for secondary resource recovery at mine waste sites. This could be attributed to the early launch of the Landsat series, which provides opportunities for many old mine waste dumps, and to the high resolution of Sentinel-2, which provides more accurate data for various applications. Sentinel-1, as an active radar satellite, offers a distinctive capability for safety monitoring related to waste stability and deformation. Besides these frequently used satellites, platforms from other groups have also been utilized, for example, the Ziyuan and Gaofen satellite series from China for safety monitoring, TerraSAR-X from Germany, and the Himawari geostationary satellite from Japan for tailings dust dispersion monitoring. These diverse sources are often integrated with Sentinel and Landsat satellite data to produce more comprehensive and accurate models. Apart from these open-access platforms, commercial satellites, such as WorldView and Pléiades, which offer extremely high spatial resolution but are limited in accessibility, are also used in some studies.

Airborne sensors can be mounted on diverse aerial platforms and operate within the Earth’s atmosphere. Among the collected articles, the unmanned aerial vehicle (UAV) was the most commonly used platform, and in the majority of the selected articles, it was the only airborne platform applied. The flexibility of these platforms lies in their ability to be applied in user-defined time frames and locations, as well as to integrate with a wide range of sensor types, enabling applications from basic mapping to high-accuracy monitoring. While many studies did not detail the device configurations, reported setups include the integration of UAV and RGB cameras, infrared and thermal imaging cameras, gamma spectroscopy, and other high-resolution cameras [97,101,107]. These configurations have been applied to a wide range of mine wastes. One outstanding usage of UAVs is the high-resolution analysis of physical properties of residuals, which are crucial parameters for dam safety and slope stability [102].

The LiDAR technique has been primarily applied for environmental monitoring in recent years. Among the reviewed articles, most application cases were from sites in North America and Europe. Its main contribution lies in the high-precision estimation of residual volumetric analysis, as well as its ability to retrieve terrain information beneath sparse natural entities when suitable acquisition settings are applied. Most LiDAR-based studies did not use LiDAR as a standalone tool. Instead, it is usually integrated with other remote sensing technologies, such as aerial imagery. The integration enhances accuracy and supports a more diverse study of parameters over time.

#### 3.2.3. Remote Sensor Applications in Mine Waste Studies

As stated above, sensors operating on different platforms were used for various research purposes or applications. These include safety monitoring (preventing accidents, structural failures, and hazards related to mine waste), environmental monitoring, and secondary resource recovery.

Safety monitoring—One of the key advantages of applying remote sensing to hazardous mine waste studies is its ability to reduce the need for on-site access. Thereby, it enhances safety not only for researchers and site operators but also for surrounding communities. This capability is particularly critical in unstable or fire-prone waste sites where traditional ground-based monitoring poses significant risks. Within safety monitoring, coal waste fire detection has been one of the most intensively studied applications. Thermal dynamics has been the primary parameter used to assess subsurface combustion activity using thermal sensors. This application has been a hot research topic in China and Poland. In an earlier study, Różański et al. [97] investigated the influence of water on coal self-combustion. They observed that the risky areas are lacking vegetation cover. A later study by Zubíček et al. [98] proposed the negative correlation between normalized difference vegetation index (NDVI) and surface temperature, highlighting the potential of NDVI as an indirect indicator of coal waste fire activity. To address the limitations of 2D imaging, 3D models generated via UAV have been developed, improving spatial accuracy and correcting 2D distortions. Although certain variations exist, UAV-based temperature showed good agreement with field-based thermal sensors [99], and the trend between NDVI and the thermal dynamics of coal waste is further confirmed.

Another major focus of safety monitoring is assessing the stability of waste piles. This includes the study of vertical settlement, horizontal displacement, seepage, construction phases, storage capacity, and many other parameters. The dominant factor in dam failure has been suggested to be related to the deformation and the water content. Remote sensing SAR and InSAR have been widely used to detect ground movements with high temporal frequency and subcentimeter accuracy. At the Sierra Minera site, a topography-driven method identified 43 active slope units among the 1959 units studied [69]. Among the active units, 21 showed significant ground movement. An increase in water content increases the risk of instability by reducing surface friction among piled particles, further intensifying deformation. In a recent study, a joint analysis method combining InSAR-derived deformation and SAR water content was developed and tested on the Majiatian and Maanshan dams in China. The Córrego do Feijão tailings dam failure was also used as a validation case. The method successfully identified a critical risk inflection point occurring around December 2018 [84].

Beyond monitoring, granular characterization of waste piles has emerged as an innovative application of UAVs with RGB cameras. The measurement is often combined with ML or other algorithms to automatically identify or predict the particle size distribution of waste piles, which is closely linked to slope stability. The result has indicated effective and efficient measures. The classification accuracy has reached over 90% on a coal mine waste dump in Australia [101]. In another case in the Caribbean archipelago, the percentage error for coarse degree prediction ranges within ±6%.

Environmental monitoring—A diverse range of phenomena has been studied through various parameters. Mineralogy and volumetric information have been the main focus. The mineralogical composition indicates the potential for AMD through the presence and abundance of specific minerals, which reflect the site’s pH. AMD develops when sulfide-bearing minerals react with oxygen and water, generating acidic run-off. Remote sensing, while enabling large-scale analysis, faces challenges due to material heterogeneity and mixed-pixel effects, in which multiple materials are represented within a single pixel. To address this, the subpixel method has been explored and shown strong performance in mapping iron levels, with a correlation of 0.76 between hyperspectral images and ground ICP measurements, as well as classifying Cu-related waste with over 79% accuracy [93,94].

Volumetric information reveals the physical size and mass of waste material. It is crucial for estimating the erosion, total contamination load, and potential long-term risks. For instance, the integration of LiDAR and aerial images quantified a total release of 10.3t of pollutants over five years in the Hiendelaencina district, Spain [112]. A significant amount of hazardous elements, As, Pb, and Zn, remain stored in the tailings pond, with potential for further dispersion [113].

Remote sensing supports monitoring in inaccessible or hazardous areas, such as underwater and radioactive sites. UAV technologies have been expanded to improve geolocation accuracy through advanced global navigation satellite system (GNSS) positioning and processing. Kim et al. [108] demonstrated the effectiveness of real-time kinematic (RTK) GNSS geolocating, and when unavailable, precise point positioning can serve as an alternative. In their real case application, relative camera location error around 27–52 cm and orthomosaic image mapping error around 102–181 cm are promising compared to standalone GNSS.

Secondary resource recovery—Satellite and airborne platforms have been used to explore the precious and critical minerals within mine waste. Different parameters have been captured to further assist the recovery plan and the determination of economic potential. Similar to environmental monitoring, volumetric information has been a critical parameter that has been frequently measured by UAVs. It provides rough estimates of grade calculation, the effort required for recovery, and the feasibility given the topography. A sulfide waste pile in Cyprus has been calculated to have a total volume of 56,000 m^3^, and another waste pile at Lousal mine in Portugal has been calculated to range from 308,478 to 322,455 m^3^ [109,110]. Using in situ portable XRF (pXRF) measurements, the latter study proposed potential tonnages of Al (24,238 t) and Zn (8272 t). The integration of remote sensing provides spectral information for mapping mineral residuals. Spectral characterization could reveal the mineralogy and geochemical concentration distribution from reflectance in the waste area and assist decision making. The SWIR spectra revealed the distribution of Sc-bearing minerals around the tailings and the washing plant [96]. Similarly, the remote sensing inversion method with ML has successfully predicted the distribution of Au at a site in South Africa, achieving an R^2^ of 0.917 and a median absolute error of 0.006.

The included studies exhibited a wide range of technical approaches and platforms. Among the airborne studies, several lack detailed reporting of sensor settings, potentially introducing uncertainty. Satellite-based studies varied in spatial coverage, resolution, and revisiting frequencies. The selection of platforms was often influenced by monitoring objectives and data availability. For instance, the geostationary satellite Himawari 8 was chosen for its constant monitoring of a waste site in Mongolia instead of the available high-resolution orbiting satellites [89]. Similarly, the sensors carried by a UAV were selected based on the parameters that need to be measured. For example, gamma spectroscopy was used for uranium waste capture, and similarly, a thermal imaging camera for emission measurement [107].

Since remote sensing analysis relies on ground validation, applied imagery is commonly selected as close as possible to the field sampling period to minimize potential seasonal impact. However, revisit intervals and cloud cover may constrain satellite selection and contribute to methodological variability across studies. In contrast, UAVs offer greater flexibility in acquisition timing, which is often closely aligned with field campaigns.

Overall, the reviewed studies demonstrated increasing use of remote sensing for mine waste management and monitoring across a diverse range of purposes. However, each platform shows advantages and limitations. Satellite systems offer stronger scalability and long-term temporal monitoring, whereas UAVs provide higher resolution and operational flexibility for site-scale investigations. Future studies would benefit from integrating remote sensing platforms and ground-based sensors into multi-source sensing frameworks.

#### 3.2.4. Integrated Evaluation of Sensor-Based Technologies

Ground-based sensors and satellite/airborne remote sensing serve each other as complementary observational systems. Each domain has distinct strengths, limitations, and operational applicability. Ground-based sensors provide higher precision and site-specific characterization, making them particularly suitable for validation and detailed material assessment. In contrast, remote sensing enables spatially continuous observations across local to regional scales, supporting long-term monitoring and assessment of inaccessible areas. The selection of appropriate techniques for specific tasks depends on several factors such as research objectives, scales, site conditions, and data availability. Table 7 provides an overview of the comparative evaluation of these sensor-based technologies.

The reviewed studies demonstrate clear trade-offs between sensor-based technologies. Ground-based sensors offer high resolution and analytical precision, but their application is often constrained by limited spatial coverage, site accessibility, and scalability. Local geological and environmental conditions may influence sensor performance and effectiveness. In contrast, remote sensing demonstrates broader coverage and higher scalability. Yet it is limited in resolution and require complex pre-processing, such as atmospheric correction [116]. The effectiveness also depends strongly on the availability of suitable imagery and temporal alignment with field validation.

Both approaches involve substantial operational and financial investment from different perspectives. Ground-based sensors often require direct access to samples, either in the field or through a laboratory, which can increase labor, travel, and maintenance costs. Remote sensing approaches involve higher computational and technological demands. Nevertheless, remote sensing generally offers greater cost-efficiency, given the service life of satellites and their broader applications beyond mining-related topics.

An integrated approach that combines ground-based measurements with remote sensing offers significant advantages for mine waste characterization. Instead of being applied separately, these approaches demonstrate stronger performance when used together through multi-source data integration. Ground-based measurements support fundamental characterization and validation of remote sensing analysis, while remote sensing extends localized observations into broader spatial and temporal contexts. High-resolution imagery may guide sampling strategies at early stages, whereas long-term satellite monitoring supports post-assessment.

Such integration enables multi-scale analysis by merging high-resolution in situ data with the broader spatial coverage of satellite imagery. Temporal trends identified from satellite data can be validated through periodic field sampling, thereby improving interpretation reliability. This approach also supports semi-real-time monitoring, where remote sensing observations can be calibrated against direct geochemical measurements to enhance analytical accuracy. Furthermore, the integrated framework supports applications such as risk mapping, predictive modeling, large-scale monitoring, and environmental impact assessment.

Despite these advantages, integrated approaches involve moderate costs and higher computational demands due to data fusion and harmonization requirements. Its effectiveness also depends on appropriate data integration, harmonization, and the application of data-driven methods. Furthermore, it may raise uncertainty concerns from data fusion, which requires careful handling of the limitations of each sensor. Overall, no single sensor system is universally optimal across all applications, and integration through data fusion represents a promising direction for enhanced characterization of mine waste properties.

### 3.3. Data-Driven Methods

Sensor-based technologies provide multi-scale, multi-variate data that are essential for mine waste management and monitoring. The high-dimensionality and complexity of these datasets require advanced data-driven methods for efficient processing, enhancement, quantification, automation, and predictive analysis. In practical applications, raw sensor measurements could be noisy and insufficient to fully represent the dynamics of mine waste systems. Data-driven methods therefore play an important role in transforming sensor-derived data into interpretable and meaningful information for monitoring and decision making. These techniques allow researchers to move beyond 1D point-based analysis toward 2D and 3D interpolation, spatial–temporal pattern recognition, anomaly detection, and predictive modeling. Such analysis serves as a critical pathway toward long-term management and monitoring.

In this section, the data-driven methods are discussed through two analytical domains: (1) geospatial methods and (2) AI-based methods, supported by geodatabases as the underlying data infrastructure for organizing, storing, and managing the complex data. The following sections introduce the results, their application, methodological characteristics, and emerging trends in recent studies.

*Co-occurrence of Keywords and Emerging Theme*

**Figure 5:**
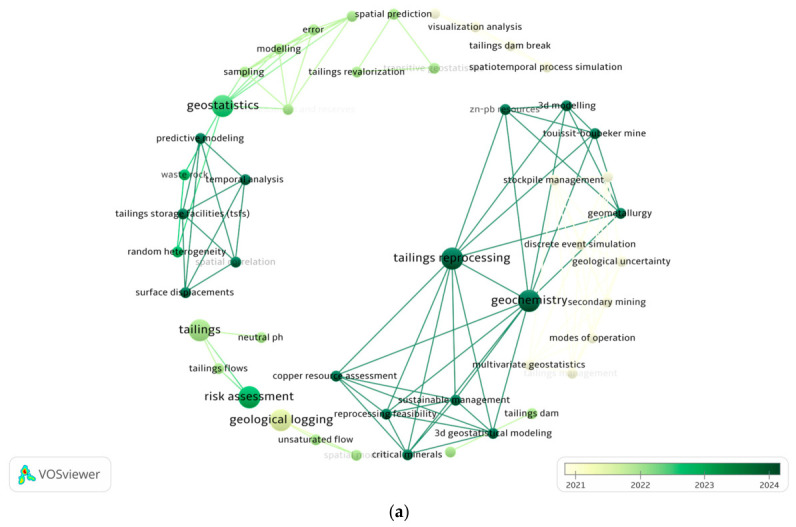

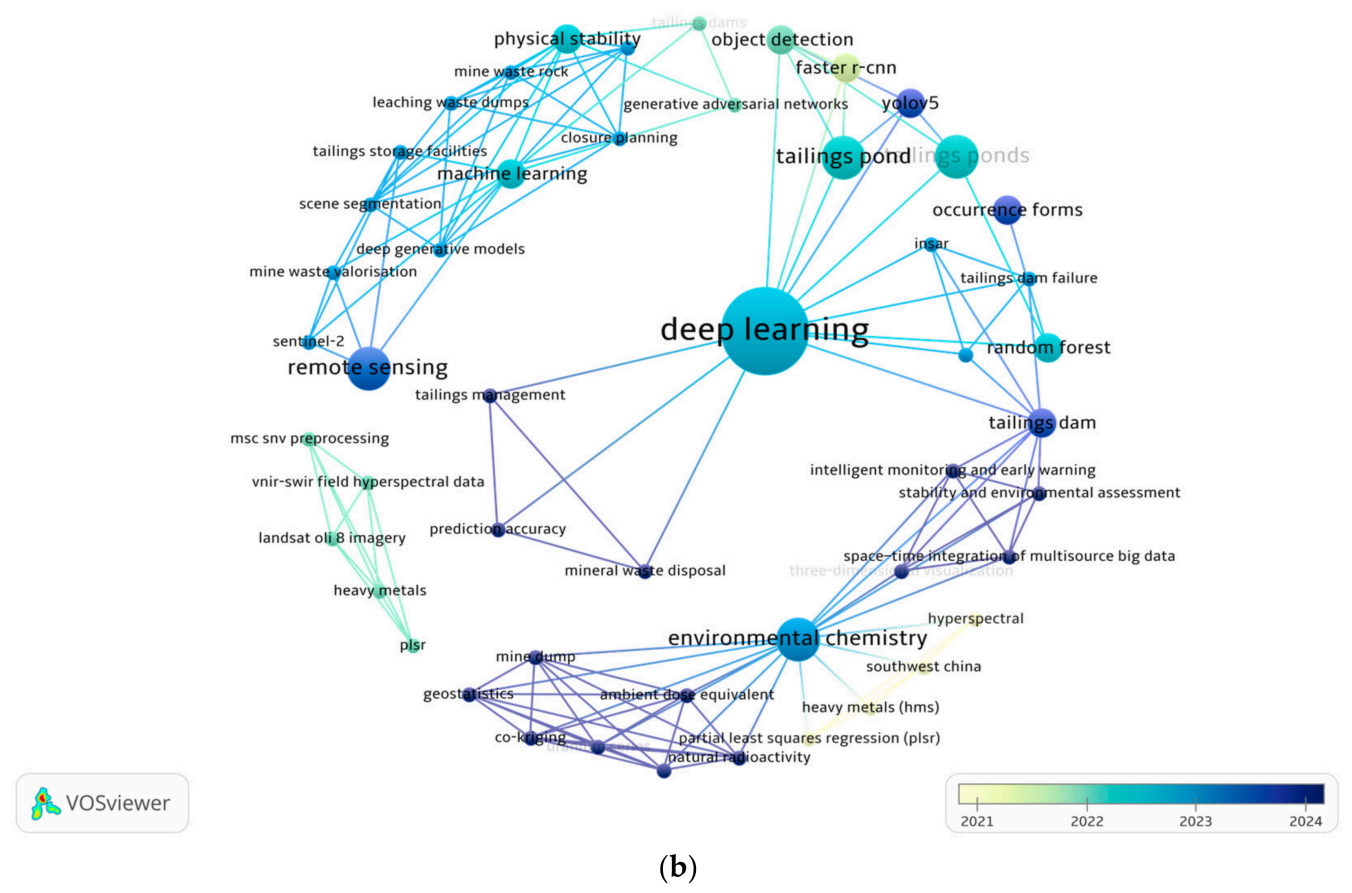
Keyword co-occurrence analysis of reviewed data-driven studies: (**a**) Geospatial-based studies; (**b**) AI-based studies.

Several frequently occurring keywords were identified for geospatial studies (Figure 5a), including “tailings”, “geostatistics”, “risk assessment”, and “geochemistry”. Similar to the results of the previous section, tailings are often the most popular study object among all waste materials. Notably, geochemistry emerged as a recent and growing research theme. This topic is often linked to studies involving the reprocessing of tailings, critical minerals, and 3D modeling, as illustrated in the graphic. In contrast, the keywords in the AI-based studies are more concentrated as shown in Figure 5b. “Deep learning” (DL) is the most frequently appearing keyword, often together with tailings ponds and object detection. In addition to AI-related terms, keywords related to remote sensing show high occurrence, alongside an emerging trend related to geostatistics.

#### 3.3.1. Geospatial Methods

Geospatial methods, from basic GIS to geostatistics, and spatial–temporal analysis have been widely applied in geoscience studies. Yet their implementation on mine waste objects remains at an exploration stage. Geospatial techniques provide a framework for interpolating, visualizing, and analyzing values at unknown points and surfaces through mathematical spatial calculations. Based on the systematic literature selection criteria, a total of 19 articles were identified that have applied such techniques. These studies mainly focus on the 2D mapping and interpolation, 3D quantification, and 4D simulation of mine waste. Table 8 summarizes these articles, including their characteristics and the main models used.

**Figure 6:**
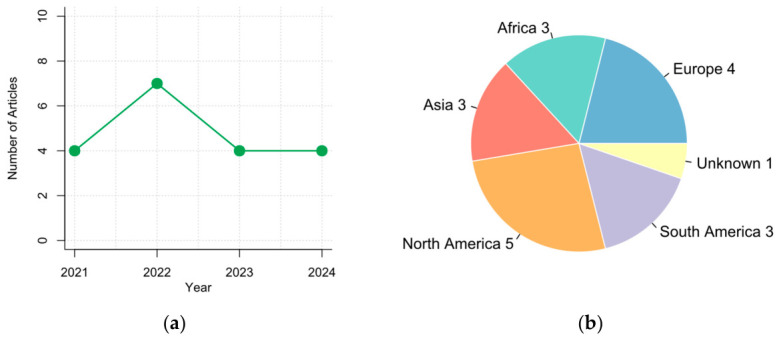
Overview of characteristics in geospatial-based studies: (**a**) Number of reviewed articles of geospatial-based studies by year; (**b**) Geographical distribution of the studied sites based on the reviewed articles.

As shown in Figure 6a, the number of geospatial-based articles included in this review has remained steady in recent years, with a small peak of seven papers in 2022. This change is mainly contributed by the exploration of the GIS environment for the visualization and simulation of tailings dam failure scenarios.

Geographically, the locations of the studied sites show an uneven global distribution, with a clear regional/country-wise clustering (Figure 6b). Study sites were predominantly located in North America (*n* = 5), followed by Europe (*n* = 4), Asia (*n* = 3), Africa (*n* = 3), and South America (*n* = 3), with one paper from Europe did not specifically mention the location of the studied site. Within each continent, the studied sites are often highly concentrated in one region. For instance, based on the reviewed articles, all study sites in Asia were located in China, and the majority of North American sites were in Canada.

These articles targeted a wide range of research purposes, reflecting the flexible role of geospatial techniques in mine waste assessment. Environmental monitoring and secondary resource recovery remain the two main topics. These two research objectives often overlap. In many cases, hazardous elements can be turned into valuable resources with appropriate treatment. Four studies focused on safety monitoring and early warning of tailings dam failure. Valorization, the conversion of waste into usable products, has been the focus of two studies. These distributions reveal not only the popular research themes, but also how geospatial methods are being applied within the broader mine waste management strategies. Overall, these trends highlight equal emphasis on monitoring and assessing economic potential, with flexible functions compared to ground-based sensors or remote sensing.

Several common limitations are recognized, which may introduce potential bias. One common problem is the lack of validation of the model performance (e.g., [117]). It is understandable in some cases that, as the used samples were collected in the last century, the “ground truth” has already experienced dynamic changes compared to the old times [118]. As a result, the modeling accuracy can only be assessed through uncertainty evaluation. Another problem is the lack of a detailed report of the sampling protocols. In some cases, sampling strategies were mentioned, but the detailed parameters were not provided. Lack of this information may create difficulties when it comes to broader comparisons.

**Table 8:** Overview of included studies using geospatial methods.

Research Purpose	Studied Location	Main Models Used	Citation
Environmental monitoring	Kenya	GIS; maximum-likelihood classification	Gitau et al., 2022 [119]
	China	Nearest neighbor index; equilibrium analysis	Hu et al., 2024 [120]
	Canada	Monte Carlo simulation; 3D variogram	Toubri et al., 2021 [121]
	Canada	Monte Carlo simulation; 3D variogram	Toubri et al., 2022 [122]
	Canada	Semi-variogram calculation	Bao et al., 2023 [123]
Secondary resources; environmental monitoring	USA	Sequential Gaussian simulation; kriging	Karacan et al., 2023 [118]
	Germany	Universal sequential Gaussian simulation	Blannin et al., 2023 [124]
	Chile	Co-kriging (CK)	Gonzalez-Díaz et al., 2022 [125]
Secondary resources	Greece	Ordinary kriging (OK); co-kriging; component co-kriging (CCK)	Bruno et al., 2021 [126]
	Germany	Sequential Gaussian simulation	Blannin et al., 2022 [127]
	Finland	Transitive kriging (TK)	Soto et al., 2022 [128]
	Morocco	Inverse distance weighting (IDW)	El Aallaoui et al., 2024 [117]
	Morocco	Ordinary kriging	Koucham et al., 2024 [129]
Valorization	Chile	Ordinary co-kriging; kernel density	Wilson et al., 2021 [130]
	Brazil	Ordinary kriging	Lemos et al., 2023 [131]
Safety monitoring	China	VGE—Computational fluid dynamics model	Yu et al., 2021 [132]
	China	GIS—ARIMA	Nie et al., 2022 [133]
	Canada	GIS—LAHARZ	Innis et al., 2022 [134]
	Not specified	Hotspot analysis; geographically weighted regression (GWR); exponential trend prediction	Koperska et al., 2024 [135]

Three distinct categories of implementation could be identified in geospatial methods. The first category is the basic spatial analysis of the residuals of their location and extent. With ArcGIS as a tool, Gitau et al. [119] used maximum-likelihood classification and TIN to map the extent and volume of an iron ore mine waste in Kenya. Their site covers an area of approximately 591,100 m^2^, and the classification accuracy was around 74%. In contrast, Hu et al. [120] conducted a large-scale spatial study of all the identified tailings within the Chinese mainland. They identified the spatial aggregation of these piles using kernel density estimation and suggested that key management strategies should include recovering potential resources rather than just preventing failures. This identified focus of the current strategy aligns with the observation from the previous section on sensor-based technology.

The second category is the 3D geostatistical modeling for quantifying mine waste. These articles mainly use the element or oxide grade as model variables and develop the distribution into 3D block models to estimate the tonnage. The basic model is IDW, which is popular in industry applications due to its fast, simple calculations. In a recent study, the IDW-based method estimated Zn concentrations of approximately 1000–20,000 ppm in the core area of the tailings, with 1200–11,000 ppm of Pb and 2–50 ppm of Ag [117]. Kriging has become a standard approach in this topic and provides a more accurate estimation [129]. Beyond the simple and ordinary kriging, several studies have explored its variations to improve spatial precision. For example, in estimating the Fe_2_O_3_ concentration, co-kriging improved the cross-validation accuracy by 0.057 compared to OK (*ρ* = 0.519), while component co-kriging achieved a further improvement with accuracy *ρ =* 0.683 [126]. When quantifying the cobalt grade, the TK accuracy correlation achieved 0.716, which is around 0.2 higher than OK [128]. Accounting for the strong heterogeneity and trend, a few studies adopted sequential Gaussian simulation. In one case study in Germany, the agreement between observed and predicted values for most of the elements and oxides has reached 68–79%, indicating a usable result [124].

The third category is the simulation of dam failure using GIS and spatial–temporal analysis to explore the early warning system. The combination of GIS environment and computational fluid dynamics model, autoregressive integrated moving average (ARIMA) model, and LAHARZ (a GIS-based program originally applied to lahar inundation areas) has shown high agreement in simulating failure scenarios from previous incidents at different locations. In a recent study, researchers employed advanced spatial statistics methods to model the stability of a tailings dam [135]. The GWR model achieved a coefficient of determination of around 0.45, indicating a moderate correlation between surface and underground displacement. The exponential trend analysis describes the temporal evolution of total displacement with R^2^ as high as 0.97, suggesting a strong exponential increase in surface movement over time. These findings highlight the importance of integrating spatial and temporal data analysis techniques into failure prevention towards a more effective early warning system.

Overall, the majority of the articles focused on the geostatistical modeling of small-scale sites and quantifying the elements of interest. The results vary across studies due to several factors during model establishment. At the initial stage, studies have implemented different sampling strategies. Combined with the physical and geochemical characteristics of the studied residual, these strategies led to varying degrees of representativeness. In the later quantification stage, researchers have used non-uniform intervals for 3D block models. Large intervals may lead to underestimation, while finer intervals may cause overestimation. However, this also depends on the sampling density, element distribution, and the interpolation methods applied. The major differences lie in the diverse geostatistical models adopted, particularly for interpolation. TK and component co-kriging have been reported to outperform ordinary kriging in certain contexts. While both OK and CCK assume local stationarity of the primary variable, CCK incorporates the spatial component that is strongly correlated with the primary variable. On the other hand, TK does not rely on the stationary hypothesis, making it more suitable for elements with high spatial heterogeneity.

Geospatial methods, as a powerful tool in processing spatial information, have mature applications in environmental studies. During the selection of articles, several papers have demonstrated advanced geostatistics applications on mine-waste-related environmental variables, for example, the use of GWR on surrounding streams [136]. Although these articles are outside the scope of this review, these algorithms could potentially be valuable references.

Overall, geospatial methods have shown promising capability for interpolation, analysis, quantification, and simulation of mine waste. Their key strength lies in transforming sparse field measurements into continuous spatial representations, enabling 3D quantification, cartographic visualization, and early warning applications. However, predictive performance remains strongly dependent on sampling representativeness, model assumptions, and the spatial heterogeneity of the waste materials. While advanced geostatistics models often outperform simple mathematical interpolation, increased complexity of calculation creates barriers in practical application. Future research would benefit from understanding model performance across scenarios and strengthening the exploration of geospatial model applications.

#### 3.3.2. Artificial Intelligence Methods

AI methods, specifically ML, and its subset DL, have enabled innovative applications in mine waste management and monitoring towards automation. These approaches have been applied to a wide range of tasks to handle complex, high-dimensional datasets with rapid processing time. In recent years, AI has been mainly used for the detection of mine waste at a large scale, the classification of mine-waste-related features, and the prediction of valuable insights. In this review, 27 articles were included that have applied ML techniques specifically to solid mine waste.

During the content screening, several studies initially appeared eligible but were excluded after the detailed review. For example, some studies applied similar sensor-based or machine learning approaches to general soil monitoring without a specific focus on mine waste or TSF structure. Although these studies provide valuable methodological insight, they were excluded as they do not directly address the monitoring of mine waste. Table 9 provides an overview of the selected articles, summarizing their characteristics in method, application, studied locations, and other attributes.

The reviewed articles employing ML methods on mine waste management and monitoring revealed several distinct application categories. These diverse applications reflect the capabilities of ML in handling spatial–temporal–spectral data derived from both ground-based and remote sensing sources:

1. Waste object identification and detection (*n* = 7):

Studies in this category use DL algorithms to automatically detect the mine waste sites, such as tailings ponds, on the Earth’s surface. The general purpose is to achieve fast and accurate positioning of waste at the border scale for better management. The You Only Look Once (YOLO) family and convolutional neural networks (CNNs) with their variations have been the dominant methods. The algorithms are mainly being applied to remote sensing sources, especially satellite imagery.

2. Waste object segmentation and extraction (*n* = 4):

Studies in this category move beyond simple positioning toward more complex calculations. DL algorithms are primarily used not only to extract waste objects themselves but also associated characteristics related to the storage facilities for safety monitoring, for example, dry beach length and rill erosion. U-Net with other combinations is the dominant algorithm for segmentation tasks.

3. Waste object classification (*n* = 3):

In this category of studies, a set of waste objects is classified into different pre-defined categories based on several parameters. The main purpose is to support decision making, for example, by classifying TSFs as healthy/non-healthy or stable/unstable. Artificial neural networks (ANNs) and random forest (RF) are commonly used algorithms for this task.

4. Prediction of characteristics (*n* = 8):

As a major application of AI methods, the regression model covers a wide range of predicted parameters related to mine waste characterization and monitoring. With known parameter values, researchers use ML to predict unknown parameters. One major group is to predict geochemical concentration and occurrence form, and the other major group is to predict physical characteristics for safety monitoring, for example, infiltration line and deformation. These regression models are commonly trained with the combination of laboratory-based sensor data, field-sensor measurements, and remote sensing data. Partial least squares regression (PLSR), RF, support vector machine (SVM), and neural networks (NNs) are some frequently mentioned algorithms.

5. Multi-purpose applications (*n* = 3):

In earlier years, studies incorporated multiple AI methods to achieve a higher level of automation. The combination of tasks includes detection and segmentation, as well as classification and regression, but they are not limited to these specific combinations.

6. Framework integration (*n* = 2):

In recent years, researchers have been developing integrated, novel frameworks for mine waste monitoring and early warning systems. These systems often incorporate remote sensing data from space and high-resolution images from airborne and terrestrial observation to provide long-term and real-time monitoring with GIS visualization. The integration of ML into such a system enables the fast analysis and prediction of spatial–temporal data, further enhancing the early warning system.

**Figure 7:**
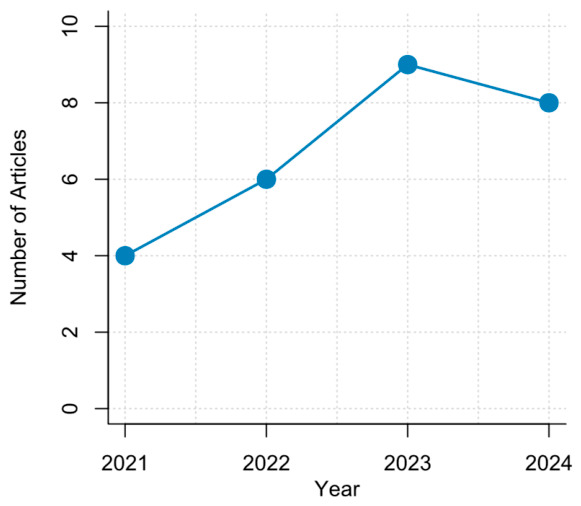
Number of reviewed articles of AI-based studies by year.

As illustrated in Figure 7, the number of publications has grown steadily over the past 4 years. In the early years, studies predominantly focused on object identification and detection. From 2022 onward, a broader range of ML applications began to emerge at more localized scales, with some of them focusing on a single tailings site. This shift of applications reflects a growing sophistication in how ML is being applied.

The reviewed articles cover a wide range of geographic regions, with noticeable variations across application tasks. In the regression and classification tasks, the studied sites were distributed more evenly across the six continents. In contrast, articles conducting the detection and segmentation tasks have mainly focused on regions in China. This pattern could reflect differences in the availability of input data.

Although different evaluation metrics have been used across tasks, the results from the reviewed articles demonstrate a promising implementation of AI in mine waste management and monitoring. Comparative analysis has been widely applied across methods to highlight the improvement achieved by proposed methods over simple algorithms.

The major motivation for implementing AI in the detection, identification, and segmentation of waste materials is that traditional remote sensing is not able to provide timely, accurate, and automated monitoring of tailings ponds at large scales. One common method in the past was visual inspection of satellite images to manually identify tailings ponds. This method obviously has a low level of effectiveness and efficiency. By implementing advanced YOLO families and R-CNN variation DL algorithms, researchers could identify the tailings from satellite images with up to 99% reduced total processing time compared to traditional ML algorithms [137]. These fast identifications remain highly accurate with average precision ranging from 85% to 99.6%. Comparison has also been made between YOLO families and R-CNN variations. At the current stage, improved YOLOv7 has demonstrated a better result in average precision metrics, where its index could reach 2 to 4 times higher than Mask R-CNN [141,142].

In addition to enabling rapid detection and delineation of tailings ponds in satellite imagery, machine learning approaches can automate the prediction of previously unknown sites using trained models. Classification models could accurately assign the study objects into categories, which assist in decision making. For example, Yu and Zahidi [149] implemented a DNN and ResNet-50 model to classify whether tailings ponds are potentially at risk of pollution. By using large-scale training data from China, Australia, and Malaysia, their optimized model could reach an accuracy above 80% based on multiple validation methods.

Regression models can be used to predict continuous variables with high precision. Among algorithms, RF has been proven to be one of the most stable methods that provides promising results where R^2^ is higher than 0.85 in multiple application cases while RMSE is relatively low [152,156,161]. Beyond the common algorithms, several articles explored advanced settings. When estimating and validating the Au concentration of two tailings in South Africa, AdaBoost has achieved R^2^ = 0.946, outperforming RF by approximately 0.06 at both sites [152]. ELM and PLSR could also achieve R^2^ > 0.80 in estimating the content of base (Cu, Zn) and critical metals (Pb, Cd, Sn) around an abandoned mineral processing plant [153].

The characteristics of the regression model make it emerge as an additional way to use geostatistics in value interpolation. Expósito-Suárez et al. [154] compared neural networks and co-kriging in estimating radiological parameters at an abandoned surface copper mine with 141 in situ measurements. While geostatistics methods show less biased results in most of the measurements, neural networks exhibit fewer deviations with lower RMSE. This suggests that ML-based methods could minimize extreme errors and potentially serve together with geostatistics to provide more robust estimation.

One of the optimization methods frequently used in ML models is data augmentation. Example experiments have been demonstrated in detection, segmentation, and regression cases. While using PSVED with augmented data, the quantitative evaluation results were improved by around 2% in precision, recall, and F1-score [145]. In contrast, the result is reduced by approximately 2% in another case study in detecting rill erosion using UAV images [146]. This revealed the improvement in increasing accuracy and optimizing model overfitting. Zhang et al. [145] presented a case study using a geostatistically augmented dataset. However, comparison analysis was not conducted as the original datasets involve confidentiality issues.

Building ML and DL models involves several steps that can introduce differences across studies. As most of the studies were applied to different study regions and different scales, differences in results are inevitable. While predicting the same parameter, studies could employ different sources of information and different pre-processing methods [88,145]. Different evaluation strategies could also slightly influence statistical results. However, such methodological variance in splitting datasets is not expected to significantly alter the overall conclusion at a broader scale. Sensitivity analysis has been conducted in many articles. Despite the grouping, the majority of improvements have been proven to be effective, especially data augmentation.

The risk of bias in the 27 included articles was evaluated. Although nearly all the studies reported the evaluation or performance metrics, overfitting is a potential risk as not all the articles split their data into training–testing–validation sets. Another potential risk is lacking transparency in the experiment data. For example, some articles did not specifically describe the location of the studied sites, and some articles only provided information at the country level [143,146]. In the detection and segmentation tasks, the study areas are predominantly located in China, with only one article focused on Chile based on the reviewed articles [141]. Although it provides an easy cross-comparison within the reviewed paper, it might introduce regional bias, which should be considered for future studies.

Overall, the effectiveness and efficiency of AI in the management and monitoring of mine waste have been proven with solid examples. At the current stage, ML and DL have been producing promising results in a wide range of applications, including detection, classification, and prediction in mine waste studies. By exploring diverse processing methods, the future capabilities and performance of AI hold great promise.

#### 3.3.3. Comparative Insights of Analytical Methods and Their Linkage to Sensor-Based Technologies

Despite the division of domains for review purposes, sensor-based technologies and data-driven methods should not be considered separately but rather as interdependent factors within mine waste monitoring systems. The characteristics of sensor-derived data vary depending on the sensor type, spatial resolution, spectral richness, noise structure, and temporal sampling frequency. These properties directly influence not only the selection and performance of analytical models but also the required data pre-processing workflows. These differences contribute to the methodological inconsistency across the studies when performing the same research tasks.

Different sensor outputs often require specific pre-processing procedures to reduce noise, correct interference and improve analytical reliability. For example, spectral data commonly require signal processing, such as smoothing, whereas geochemical measurements may require transformations to reduce element skewness. Similarly, remote sensing imagery often involves radiometric, geometric, and atmospheric corrections to obtain the surface reflectance that are comparable to ground-based sensor output. Such pre-processing steps could significantly influence further analytical performance and comparison across studies. Differences in algorithms and normalization may directly influence model sensitivity and outputs.

High-resolution and high-dimensional datasets, such as hyperspectral imaging, are often better suited for ML and DL approaches because of their ability to capture complex and non-linear patterns from large-volume data cubes. In contrast, sparse or noisy measurements may require geostatistical or probabilistic modeling techniques that reduce uncertainty. Similarly, multimodal datasets derived from multiple sources may benefit from data fusion approaches and hybrid analytical frameworks, allowing complementary information to improve robustness and predictive performance.

The reviewed studies suggest that geospatial and AI-based methods are being considered as both complementary methods and “competitors”. Geospatial methods can handle uncertainty effectively by applying probabilistic estimation and accounting for spatial autocorrelation. In contrast, ML models study the pattern from the available data and, therefore, their uncertainty handling largely depends on selected model structure, training settings and data quality. Certain ML approaches, such as Gaussian processes, may also provide robust uncertainty estimation capabilities. AI-based methods may also offer greater scalability for large and high-dimensional datasets, although they often require higher computational resources and large-volume input [7].

Despite partial overlapping in functions, supporting evidence comparing the capabilities of geospatial and AI methods remains limited. Existing literature demonstrated that these two domains produce comparable results when transforming 1D measurements to 2D surfaces [154]. However, broader performance differences remain uncertain, and whether one approach consistently outperforms the other across different tasks is unclear. Further efforts are necessary to strengthen the understanding of trade-offs between these two techniques.

These findings suggest that the selection of appropriate data analytical systems is fundamentally constrained and guided by sensor characteristics, data quality, and research objectives. In addition, both geospatial and AI-based methods are influenced by site-specific conditions. This nature limits direct comparison and broader transferability. Therefore, the optimization of mine waste management and monitoring frameworks should consider joint optimization of sensor data acquisition and data analytical design.

#### 3.3.4. Geodatabase

Within the scope of data-driven methods, the development of geodatabases for mine waste is emerging. Among the collected articles, four studies have explored this topic at different scales and from different perspectives. Three of the articles established geodatabases to manage and integrate spatial and attribute data from various sources, while one article systematically reviewed existing global databases related to TSFs. These geodatabases serve as foundational infrastructure for storing, querying, managing, and analyzing the spatial information of TSFs at large scales, providing a macro-level picture for decision making. The characteristics of the selected articles are summarized in Table 10. Although several studies also implemented similar methods in detecting and identifying tailings facilities, they did not further develop the results into a structured database. Therefore, these articles were categorized by their main focus and are discussed in other sections.

The included studies developed geodatabases at different geographical and administrative scales. Two articles published in 2022 focused on Australia at the national level, while others examined provincial or global scales. The scale of the database development was closely related to the source of data. Sarker et al. [164] combined visual inspection of satellite images with publicly available datasets for partial validation. Gomez et al. [165] and Mpanza [166], in contrast, primarily relied on public information gathered from the literature and requested from government departments as the major source for establishing their geodatabases.

Using visual inspection, Sarker et al. [164] have identified 331 active and 759 inactive TSFs in Australia. However, complete validation across the entire study area was not possible due to the limited data availability. Gomez et al. [137] compiled a dataset based on samples from seven mine residuals in Australia and estimated the concentration of several critical elements to provide an overview. Such a dataset demonstrates how geodatabases can incorporate both inventory information and geochemical attributes.

At the global scale, Massignan and Sánchez [167] conducted a systematic review across 36 mining countries and identified 12 datasets spanning over six countries. The reported datasets varied considerably in structure and information coverage. The authors acknowledge potential search bias due to language and accessibility limitations.

Overall, while the scopes and database structures differ, these studies illustrate the current efforts to develop structured mine waste inventories and highlight variability in scale, data sources, and information completeness.

## 4. Discussion

The management and monitoring of mine waste are complex and challenging tasks that need multi-scale understanding of the waste system. At the microscale, the mine waste could exhibit significant spatial heterogeneity within the waste volume. This variation can be attributed to differences in factors such as ore type, geochemical concentrations, mineral associations, particle-size distribution, and weathering conditions. On a macroscale, mine waste piles appear significantly different across environmental settings, geological zones, mining exploration methods, climate conditions, and many other factors. This review systematically summarizes recent advancements in the application of sensor-based technologies and data-driven methods for the management and monitoring of mine waste. Despite recent advancements in technology and methods, certain challenges remain to be addressed.

### 4.1. Insights on Sensor-Based Technologies

#### 4.1.1. Insights of Ground-Based Sensors

Various applications at different waste sites have demonstrated the high effectiveness of ground-based sensors, such as VNIR-SWIR sensors, in characterizing mine waste, offering insights into mineralogical, geochemical, and physical properties. The detailed characterization of waste assists in selecting appropriate treatment, further upcycling, the validation of newly emerging technologies, and could even accelerate environmental licensing at an earlier stage [3,59,94,168]. The information obtained from sensors not only facilitates environmental monitoring but also supports valorization and broader decision making throughout the management process.

The increase in recent studies with advanced technologies indicates that actions have been taken to understand how these residuals are being transformed into valuable materials and reduce their environmental impact. Tailings and other residuals, which would normally be considered non-economically viable, have shown significant potential at some locations. The recovery of these hidden resources must balance the operational, economic, and environmental costs. Although these residuals often contain relatively low concentrations of recoverable elements, they benefit from being exposed above ground and finely processed, offering a cost advantage over primary ore extraction [45,47].

Despite the advances, several gaps have been identified in current research trends. Many of today’s sensors are portable and can, in theory, be used in field environments. Yet the implementation of field measurement remains relatively rare, and the common practice is still the use of portable sensors in laboratories [169]. One possible reason is the operational safety concerns associated with radiation-based sensors. For instance, the pXRF analyzer is designed to be safe if the user follows the instructions. However, some scattered X-rays cannot be completely shielded. As a result, the use of such equipment is often strictly regulated in many countries. Operational licenses linked to specific places and the presence of certified staff are mandatory. These facts may limit the broader implementation of such technologies for on-site measurement. Even for non-radioactive equipment, transportation poses a potential risk of instrumental damage and possible measurement errors. These factors highlight the need for robust sensor technologies that can operate in harsh environments. Despite the challenges, the use of portable sensors for in situ applications could offer several advantages, including real-time monitoring, rapid data collection, cost-effectiveness, and minimal disturbance to the sampling environment. An increasing number of studies have demonstrated the use of portable systems in situ. For example, a recent study published in 2025 has demonstrated the successful in situ application of portable XRF in Portugal, reporting stable measurements and good agreement with laboratory analysis results [170]. Further expansion of portable sensor applications in field settings is therefore essential to fully realize these benefits.

Another noticeable gap in current research is the focus on different types of mine waste. While tailings have received considerable attention across various research fields, studies on waste rocks and slags remain very limited. One possible reason is that tailings are the major waste stream throughout the entire mining process [171]. Due to their finely ground nature and large-area occupation, tailings are more prone to oxidation, which significantly increases the risk of AMD formation [172]. Moreover, tailings could contain high concentrations of metals of economic interest, as they are processed ore. In contrast, slags are considered relatively more stable [27]. However, a recent study found that waste rocks at a gold waste site exhibit higher contamination levels and greater leaching potential than tailings or ores [25]. Similarly, slags are often considered “harmless” and have been widely used for construction materials. Yet an agreement suggested by recent studies is that, despite their stability, these materials can be complex, may still contain high levels of toxic elements, and may still pose long-term environmental problems [32,173]. Appropriate treatment should be applied to protect the health of surrounding communities. These findings highlight the need for further research leveraging sensor technologies to assess the mineralogical, chemical, and physical properties of waste rock and slags, especially at sites where toxic chemicals were used in metallurgical processes. Emerging imaging techniques, such as X-ray computed tomography (CT), may further complement the sensor technologies involved in the reviewed articles by enabling non-destructive 3D characterization of the internal structure of mine waste. Recent studies have demonstrated the potential of CT imaging for characterizing physical properties of tailings, waste rocks, and mining-related materials [174,175]. Such approaches could support improved stability assessment and better understanding of processes that may impact long-term environmental risks.

Modern ground-based sensors could provide accurate data for characterizing the composition of mine waste. Understanding the properties of these materials is the fundamental step toward their further application in technologies, environmental monitoring, and valorization. Consequently, such sensor technologies are increasingly being applied to previously uncharacterized or poorly documented sites, particularly historical legacy deposits [176]. In certain cases, investigations have been extended to tailings preserved in underwater environment [177]. These examples demonstrate the expanding scope and adaptability of ground-based sensor technologies.

#### 4.1.2. Insights of Satellite and Airborne Remote Sensing

While ground-based sensors offer precise information, their coverage is inherently limited to small-scale, mainly point data from specific sampling locations. Remote sensing technologies, including but not limited to satellite and airborne platforms, have emerged as powerful tools for large-scale mine waste management and monitoring. These methods bring several advantages over traditional field approaches, particularly in terms of spatial–temporal resolution, cost-efficiency, safety, and operational scalability. Additionally, while ground-based sensors are primarily used for characterization, satellite and airborne remote sensing technologies can be implemented for various purposes, including monitoring, detection, and characterization to achieve 4D analysis.

Each platform and attached sensor offers distinct advantages. Satellites allow long-term monitoring and extensive spatial coverage. Airborne devices provide flexible and high-resolution imagery and can be adjusted depending on the research need. The temporal resolution could be as high as desired with daily feedback [178]. For example, LiDAR is a good choice for terrain and volume analysis. It offers 3D topographic data and the ability to penetrate surface and vegetation coverage, which are inherent limitations of satellite and aerial images. A growing trend involves the integration of platforms with ground-based sensors and with ML to achieve automatic characterization [73].

The UAV device was originally used for the military. With recent advances in geolocation methods, the use of UAVs could be extended to remote sites where virtual reference station (VRS) infrastructure is unavailable, which is highly possible for historical waste sites. The recent experiments of applying UAVs for physical characterization also demonstrated the effectiveness of airborne remote sensing at the site level [179]. Yet, several limitations in implementation persist despite steadily growing interest, such as restrictions on drone operations.

UAVs are usually subject to prohibited flying areas defined by aviation authorities for safety and operational reasons. Waste areas could possibly be covered and, therefore, the “access” to the site remains limited compared to open-source satellite images [102]. While studying large waste areas, one limitation that needs to be considered is the battery life used to support the UAV. Although, with a low flying height, UAVs may not be affected by weather with extensive cloud coverage, they are still limited by precipitation, wind, and mist. At locations where abrupt changes of terrain exist, the flying of UAVs may also introduce large errors and expose them to falling risk [103,180].

The cost-effectiveness of remote sensing technologies can vary significantly across contexts. While some studies proposed that aerial LiDAR is more affordable than UAV implementation, others claim the opposite, where a UAV has been chosen because of the cheaper cost compared to LiDAR [103,113]. This difference in argument could arise from data acquisition stages, regional operational costs, existing infrastructure, and research frequency. For example, designing, building, and maintaining a satellite requires vast investments in cost and time. However, once it is successfully launched and operational, it provides long-term benefits, with valuable, readily available data sources for diverse research fields. This is also true for airborne platforms, especially UAVs, even when considering the training of UAV operators [107]. Despite the discrepancy, the consensus is that remote sensing methods have a higher cost-effectiveness ratio than ground-based sensors and physical sample measurements.

Safety monitoring has been the key research purpose in recent years with the use of remote sensing. The high-frequency representation of this category could be driven by the strong concerns from society about human life, infrastructure, and the ecosystem. These hazard incidents usually require long-term monitoring across a wide area, making in situ measurement challenging or impractical. Remote sensing offers promising solutions through its ability to provide frequent surveillance and timely updates, especially with satellite platforms. This is particularly valuable for early warning systems. In contrast, environmental impacts often occur gradually, and the exploration of valuable resources from waste often requires ground-truth validation to secure investment.

In response to these monitoring demands, various methods and parameters have been developed to capture the early signals of potential failures. False alarms have been a concern in this context. Failure of waste piles could release heavy metals or PTEs into the environment. However, it is difficult to assess the alarm’s accuracy. The method developed by Yan et al. [84] has successfully identified a waste dump with potential failure risk. Actions have been taken until the indicator becomes “safe”. Nevertheless, it remains uncertain whether failure would have occurred without intervention. Although the method identified a turning point prior to a major failure event in Brazil, the limited sample size, two cases with one actual failure, introduces a high likelihood of coincidence. The dilemma is that treating risky dumps requires an investment of money and time. The limited budget should be allocated to the priority piles that are at high risk. Yet the cost of ignoring the potential risks in the trial could be human lives. This requires researchers to keep on developing highly accurate predictions and alarm systems. The incidents that happened in the past could serve as valuable references. Only tracing back after the hazard has happened is unacceptable.

One of the key challenges in volumetric studies using remote sensing is the lack of historical terrain data. Numerous mine waste dumps were created long before satellite imagery or terrain documentation was available. As a result, the original ground surface is no longer traceable. This nature makes it difficult to accurately estimate the waste volumes of the materials over time. Various computational approaches have been implemented to obtain rough estimates, but without a known ground elevation, such estimates are largely comparative and lack validation. This requires the study of waste sites where the original topography is available to validate the accuracy of methods. It also serves as a critical reminder of the need for documentation in ongoing and future mining activities.

To address the current limitations and expand the usability of remote sensing, several promising directions have emerged. One of the methods is the integration of multiple remote sensing platforms. As the conditions of the waste sites vary, combining multiple remote sensing datasets could enhance the temporal resolutions and help capture dynamic and key processes [90]. One inherent characteristic of remote sensing images is their pixel-based nature. The spectral reflectance within a pixel could possibly be from different minerals. Techniques such as pixel unmixing have shown promise in addressing this issue, but further exploration and validation are needed.

Finally, the reviewed studies indicate some variation in the application of remote sensing techniques across regions. This pattern may be partly associated with data, resource requirements, and the accessibility of infrastructure. The feasibility of applying airborne techniques may be difficult due to the site-specific conditions, such as environmentally sensitive areas and limited connectivity [107]. These observations suggest that communication and collaboration efforts could potentially support a broader application of advanced technologies.

### 4.2. Insights on Data-Driven Methods

#### 4.2.1. Insights of Geospatial Methods

GIS, geostatistics, and spatial–temporal modeling have become core methods for understanding the spatial variability within mine waste and the environmental impacts. As discussed in previous sections, it is often impractical to obtain field samples across the entire study area due to cost and time constraints. In this context, integrating geospatial methods with ground-based sensor data provides a more efficient and cost-effective solution. Such integration enables more accurate estimation of the resources within the tailings that show heterogeneous composition and spatial distributions. This information could further support and guide the reprocessing of secondary resources [181]. However, at the current stage, there are a limited number of articles compared to the other techniques.

One ambiguous aspect is the adequate number and spatial distribution of sampling points required for constructing reliable geostatistical models. In this context, the design of the sampling strategy plays a critical role in ensuring accurate and representative resource estimation [181]. Studies such as Blannin et al. [127] and Bao et al. [123] have both devoted efforts to comparing sampling strategies. The results from the two studies offer complementary insights, while some of the conclusions are in agreement. Blannin et al. [127] specifically compared the effectiveness of random sampling and grid-based sampling, as well as the minimum number of samples required for accurate models. They suggested that a relatively low sampling density is sufficient for the assessment and the combination of a regular grid and random points could potentially be a best practice. Bao et al. [123] also suggested that only using systematic sampling may not capture the randomness of elements. Yet their result indicates a higher density of samples may be necessary. As mentioned by Blannin et al. [127], the sampling design should always account for the historical process of waste generation. In addition, variations in chemical and mineralogical characteristics across different waste types may influence sampling outcomes. As demonstrated by Wen et al. [25], different types of waste can vary significantly in their composition and form. However, a comprehensive understanding of how these differences affect optimal sampling strategies remains limited, highlighting the need for tailored methods at each site. Therefore, further comparison studies are required to improve the selection and design of sampling strategies, especially when dealing with diverse types of waste.

Robust models are essential for accurately assessing the economic potential of elements in mine waste. The target elements often occur at low concentrations, as highlighted in several ground-based sensor studies discussed earlier. Current geospatial models for interpolating element concentrations generally demonstrate strong predictive performance compared to conventional methods, such as IDW. However, the range of geospatial techniques applied and the application for which they are used remain relatively limited. In previous and recent studies, IDW and kriging and its variations have been the most frequently used algorithms. While, in some cases, fundamental kriging may produce better results than complex models considering the characteristics of the mine waste, many advanced spatial modeling methods are unexplored with unknown effectiveness. A recent article published in 2026 introduced the multi-Gaussian co-kriging (MGCOK) method, which explicitly accounts for strong inter-correlations between elements within the residual [182]. The incorporation of secondary variables in modeling has demonstrated improved performance. Such approaches provide valuable references for mine waste studies as elements often exhibit strong correlation at many sites [180]. Beyond interpolation, geospatial approaches also offer opportunities for more comprehensive spatial–temporal analysis and visualization to support decision making. However, their broader potential in mine waste applications remains insufficiently explored.

#### 4.2.2. Insights of AI-Based Methods (ML)

The application of ML and DL has demonstrated great potential in advancing waste management and monitoring. The integration of AI techniques marks a shift toward automating workflows, facilitating the transformation of data characteristics from traditionally sparse field samples to the big data era. These techniques improve predictive accuracy, accelerate data processing, and enable the capture of complex relationships among multiple parameters, thereby providing valuable support for applications such as secondary resource recovery, environmental assessment, and safety monitoring.

An inevitable challenge for implementing AI in mine waste topics is the dependency of models on numerous training data. However, with the inherent problems in traditional sampling, it is often difficult to provide sufficient high-quality data as input. The impact of training data on model performance has been reported by several studies. In a recent ML study on ore classification, Fang et al. [183] have proposed that reducing the training set has a negative impact on the classification accuracy. Additionally, big data brings the advantage of denoising by allowing averaging nearby points to improve resolution. This is usually not possible in traditional field measurements. Moreover, using remote sensing time series as big data input, the observation of seasonality and anomalies of mine waste becomes possible. This is an essential theme, especially for safety monitoring, which is difficult to achieve with the current in situ sensor settings.

Researchers have been implementing data augmentation methods to improve model performance, especially in DL experiments. Data augmentation keeps the original characteristics of data but increases the size and diversity of the training data. This method not only increases data abundance but also detects potential overfitting in models. The common implementation includes the rotation of images, color augmentations, flipping, blurring, and algorithms like generative adversarial networks (GANs) [141,142,145,148].

Yet data augmentation is not a perfect solution. Similar to all big data methods, the increase in data abundance will offset the data accuracy to a certain degree. None of the current models could achieve perfect predictive precision, especially in regression tasks when mapping the concentration of elements. A balance between these two parameters needs to be considered from several aspects. Firstly, even if a small area is identified, processing the entire waste pile or region is usually required. On one side, mine waste could occupy hundreds to thousands of hectares and store tens to hundreds of millions of cubic meters of waste material. With its cast scale and complex topography, it is not possible to conduct highly localized extractions across the entire waste area in real-world operations. Moreover, reprocessing strategies must address not only the recovery of economically valuable materials but also toxic element treatment to support environmental remediation. Secondly, the enrichment of data could build bridges between mine waste topics with more possible subjects and attract qualified personnel to bring innovative methods into this field. AI and geospatial methods are two examples. When the amount of data is enough to be implemented in algorithms, the research obstacle for trained data experts could be significantly reduced while the exploration of advanced models is already complex and difficult [152]. This improvement would increase the communication across disciplines and bring new vitality to the mining subject.

Similarly to geospatial methods, ML is not a method that is limited to mining topics. As a powerful theme in AI, they have been successfully applied in other domains before being applied to mine waste studies. One opportunity in this topic is to draw on valuable references from other research themes, especially those that share similar steps. Fang et al. [183] developed two specialized algorithms, trans-long short-term memory (LSTM) for rough mineral sorting, and RNN-overhead-XGBoost for fine sorting. Both algorithms were based on X-ray absorption spectroscopy and aimed at high-speed AI mineral classification. The classification accuracies of 93.49% and 99.21% demonstrate promising performance in ore sorting. Many ML and spatial analysis algorithms were developed for purposes beyond mine waste management. It is worth noting that, although the ore classification and waste recovery are at different phases of the mining cycle, sorting is a fundamental step in both scenarios. The key idea is to separate the valuable component from non-valuable materials. Therefore, considering this shared principle, these methodologies could be adopted for mine waste recovery.

Currently, there is a lack of algorithms specifically tailored to each scenario in mine waste management and monitoring, such as effective physical–chemical characterization and risk assessment. Thus, there is a need for the development of advanced methods. While existing models have already demonstrated promising results, it is observable that the potential of AI in this field is far from fully realized. The integration of these techniques in mine waste research offers substantial opportunities for further exploration and innovation.

#### 4.2.3. Insights of Geodatabase

Mining waste is produced at a rate exceeding 100 billion tons per year worldwide today [184]. However, detailed and harmonized information of these residuals remains limited. The findings suggest the limited availability of comprehensive and publicly accessible geodatabases for mine waste at the global level.

Although researchers have devoted efforts to establishing a database of general mining areas at the global level [185], the number of TSFs cannot be directly derived from such inventories. This is because TSFs may not be spatially adjacent to the pits. In some cases, multiple mining sites share the same TSF. In addition, many mine sites are linked to productions that do not generate tailings during processing and pose a low-level impact on the surrounding environment [164].

One of the key challenges is data availability and accessibility. Mine waste data are often owned by private companies and may be commercially sensitive. Consequently, national and provincial databases rely heavily on incomplete public records, as reflected in recent studies [165,166]. In addition, discrepancies in reported TSF numbers across available databases raise concerns regarding data reliability [164]. Even when databases exist, validation remains difficult [167].

Visual inspection is a commonly used method in establishing mining-related databases [164,185]. This method reduces the need for specialized technical expertise. However, although often referred as “remote sensing”, it does not involve advanced algorithms and is often time-consuming. Furthermore, the complex interaction between mine waste and the surrounding environment could alter the visual characteristics of waste deposits, making them difficult to reliably identify in satellite imagery.

A potential improvement method could apply ML to detect tailings and use visual inspection to check the omitted ones. However, algorithm performance depends on training data availability, and many critical attributes still require site-level information that may not be accessible.

The status of the existing databases remains unclear. A Chinese report published in 2018 by the Green Data network claims that it has identified 14,217 tailings within the Chinese mainland [186], and a searchable online database is publicly accessible. This database was identified by using the same search strategies described in the previous study, however, it was not captured in the survey conducted by the authors [167]. This indicates that both language barriers and reliance on region-specific search engines may contribute to bias in the identification of relevant databases.

The existing database of mine waste could serve as a reference for the omitted piles when integrating comprehensive information, as well as reducing the effort required to mitigate repetitive details. Such information could help allocate limited funding more effectively and prioritize exploration targets [187]. This gap appeals to further investigation of available databases for global communication.

The lack of standardized international guidelines further complicates database development. Key stakeholders of mine waste assessment include local communities, governments, mining companies, academia, research institutes, investors, and other organizations. Different stakeholders expect different information to be provided. While some databases focus on only active TSFs, some databases, in contrast, only disclose inactive TSFs [167]. Nevertheless, all the databases pose a weakness in risk communication and in how to respond to emergencies for the local community. A comprehensive database requires well-established regulatory frameworks to ensure that the contained information considers the needs of the intended audience and provides accurate, up-to-date, and easily understandable details.

A well-established geodatabase could enable a wide range of analyses at broader spatial scales and greater efficiency. However, the effectiveness and reliability of advanced spatial analysis may be constrained when datasets are sparse and incomplete. The global demand for resources continues to rise, as well as the concern related to their extraction, including associated hazards and environmental impact. National and international efforts are encouraged to prioritize the development of systematic, high-quality, and comprehensive geodatabases of mine waste.

### 4.3. Limitations and Opportunities

Mine waste is inherently complex, especially given its multiple pathways of interaction with the environment. Within the residuals, coupled geochemical processes and microbio-communities can interact and form feedback cycles that influence the residual properties over time. These wastes are dynamic in nature, for example, they are influenced by water, air, and many other parameters. These complex characteristics bring challenges and limitations to the management and monitoring of mine wastes.

#### 4.3.1. Accurate, Simplified, and Transferable Modeling

One limitation of both geospatial techniques and AI algorithms is the challenge of developing simplified yet accurate models or workflows that are transferable to other sites. Some authors acknowledge that their models may not be applicable at other sites [182]. While some studies propose that their methods could be applied to other residuals worldwide, only a few studies have tested transferability in practice. One example is the study by Zhang et al. [7,152], which attempted to test the developed remote sensing inversion method with ML algorithms to predict element concentration on nearby tailings. However, when the approach was applied to another tailings area, the available satellite imagery was captured very close to the reprocessing date, meaning recent human activities may have influenced the surface conditions and affected the evaluation of model accuracy. Even if two tailings have the same mineral products at the exploration stage, recovery difficulties could vary due to the processing methods and environmental setting [48]. In this case, how researchers should select the best practice during the sampling, pre-processing, modeling, and prediction remains challenging. Systematic studies that include many variables would be necessary, yet highly complex. Such work demands collaborations from cross-disciplinary experts and well-coordinated data acquisitions.

Moreover, although complex models often achieve more accurate results, it can be difficult for researchers to implement and interpret if experts from specific fields are not involved [124]. The heavy computation also leads to high energy consumption hidden behind the visible budget, which should be considered in ethical research. Therefore, while transferability needs to be analyzed to identify the most effective algorithms, novel methods must be further explored.

#### 4.3.2. Advancing Real-Time Sensors and Enhancing Long-Term Monitoring

A significant gap identified in this review is the scarcity of long-term temporal studies. Although remote sensing is inherently capable of time series analysis, few studies in this review have taken advantage of this technology, and most of the articles appear to be on the theme of safety monitoring—by using InSAR for dam failure and deformation prediction. For instance, Surrette et al. [38] is one of the only studies among the reviewed articles that characterized samples collected over multiple years. Gürtekin and Aydar [26] emphasize the importance of temporal observation in characterizing the properties of tailings, especially when they contain sulfides. This is because of the formation of secondary minerals. The unoxidized tailing samples analyzed in the past may not represent the actual behavior in a longer time frame. As shown by Sapkota et al. [22], the investigated cemented crust formed on top of tailings from remediation in 1970 has experienced precipitation. The sand cover originally used to stop tailings erosion has now become a new source of As pollution. This result highlights the importance of long-term observation even after the waste piles have been remediated.

Real-time, in situ sensor technologies can help address some of these temporal gaps by enabling continuous monitoring of the dynamic processes within mine waste. Recent efforts to develop in situ sensors for measuring solids content in fluid fine tailings show promise for cost-effective monitoring [188,189]. However, practical deployment remains limited due to challenges such as sensor robustness in harsh environments and calibration drift over time. These limitations highlight the need for robust in situ systems that could operate reliably under harsh conditions.

Beyond technological considerations, an implication that could be derived from the temporal abundance of mine waste data is the documentation of active or future mine waste sites including TSFs. In the reviewed articles, studies focusing on active mines are very limited. The constantly and rapidly changing landscape and anthropological activities make the characterization difficult. Additionally, the documentation may raise confidentiality concerns, as it often contains sensitive geological, environmental, operational, and economic data. However, even if sharing the data is not feasible in real time, having such plans at the initial stage could potentially benefit industries in obtaining licenses, as well as the recovery of secondary resources from residuals.

#### 4.3.3. Regulations and Practical Constraints

In the current stage of mine waste management and monitoring, one of the major challenges is the regional and global regulations. While each mine waste is unique due to site-specific factors, the various standards in different countries make the systematic transfer of management and technology experience even more difficult. In some regions, assessments of tailings resources remain restricted to research programs, with limited access to other stakeholders. Although not directly referenced, previous research indicates that the recovery of valuable metal resources from flotation tailings in Poland may be restricted by the laws at the time [48]. In a recent study, the authors also noted that numerous legal issues are associated with mine waste exploration [187]. This may also explain why in some studies the terminology of “potential resources” is used. Additionally, cooperation across countries often faces challenges due to regulatory restrictions. For instance, some waste samples may not be allowed to be transported through customs due to the elements contained in the samples and other potential safety reasons [107]. This significantly limited the feasibility of lab-based analysis for accurate measurements.

Similarly, implementing advanced technologies may be difficult due to the sensitivity of geopolitical information. Unlike open-source satellite images, UAV technology has a military application background. This character gives it stricter control in many regions. In an experiment in central Asia, researchers from European institutions encountered the problem that the operator of the UAV needs a local nationality [107]. Thereafter, they trained local experts to fly the drone. This has created significant technical barriers in countries with limited economic capacity, infrastructure, and technical experts. Researchers should respect the local regulations and therefore pre-investigation of such policies is necessary. This process could also ensure research efficiency, especially when it comes to cross-country studies.

Besides regulatory and technical barriers, the implementation of advanced technologies may also be constrained by economic and operational accessibility. Techniques such as UAVs, hyperspectral sensors, and submeter-resolution satellite imagery often require substantial investment in equipment, maintenance, infrastructure construction, and operational training. Similarly, advanced data-driven methods require high computational capacity and expertise across multiple disciplines. These requirements may limit the wider adoption of advanced techniques. This challenge highlights the importance of improving the accessibility of open-source data and developing more transferable, cost-effective workflows.

#### 4.3.4. Limitations of This Review

This review synthesizes findings from recent journal articles. However, several inherent limitations should be acknowledged. First, to ensure an accurate understanding of the content, this review applied an English-language filter. As a result, studies published in other languages may not have been captured, including those presenting advanced techniques from countries where English is not an official language. Second, although some articles appeared highly relevant based on their titles and abstracts, full access was not available at the time of the review. These papers were not included in the systematic quantitative analysis yet may still represent valuable contributions to the field. Third, this review spans a diverse range of sensing technologies and data-driven methods. In practice, many of these methods are integrated within the same workflow rather than used in isolation. For instance, remote sensing studies often rely on laboratory-based measurements as validation, while GIS approaches may incorporate ML for prediction and remote sensing data for spatial analysis. Although the classification framework was introduced for organizational clarity, the overlap among methods makes strict categorization challenging. Assigning studies to a single category based on their primary contribution may introduce subjective bias. Finally, although the review identifies trends across each thematic technique, these trends may not fully capture the complexity and progress of current research. More focused reviews on specific technologies or methodological themes would provide deeper insight into their development and applications.

### 4.4. Environmental and Social Considerations

Environmental and social considerations in mine waste research and the application of related technologies have been rarely discussed in academic publications in recent years. While significant advancements have been made in sensor-based technologies and data-driven methods, their applications raise several challenges, particularly regarding privacy, data transparency, and social responsibility.

#### 4.4.1. Privacy

The social challenge in privacy arises from the conflict between environmental monitoring needs and the rights of individuals and companies. The use of remote sensing technologies enables large-scale and high-frequency monitoring of key environmental parameters. However, they may unintentionally capture personal information, especially where the waste site is close to residential communities or within active mining operations. With high-temporal-resolution satellites, the activities of residents could be inadvertently recorded. Similarly, some waste sites remain under company ownership even after exploration and are therefore restricted in access. Using remote sensing to reveal their location, morphological changes, and characteristics might violate corporate confidentiality. In these cases, researchers must ensure the remote sensing data are sourced from open-access platforms and the disclosed information aligns with publicly available company records or other official reports. Furthermore, the balance between trade secrecy and data transparency needs to be carefully managed. While companies have legal and rational concerns about property information, the rights of the local communities, especially vulnerable groups, to understand potential pollution and disaster risks should be prioritized in research practices.

#### 4.4.2. Data Fusion

The integration of multiple data sources has been an emerging trend in the management and monitoring of mine waste. By integrating data from various ground-based sensors and remote sensing platforms, researchers can obtain a more comprehensive and accurate characterization of mine waste. However, challenges such as data compatibility, calibration, and bias must be addressed to fully realize these benefits. Each sensor has its own limitations in measurement. When these sources are combined, errors from one data type can be magnified or misrepresented, leading to incorrect conclusions about the condition of the mine waste or the environment. Recent studies have shown that proper calibration can improve the high-resolution data accuracy [190]. To mitigate data fusion problems, calibration, rigorous data harmonization, and uncertainty quantification are necessary to ensure that the fused datasets remain robust.

#### 4.4.3. Environmental Footprint

The recovery of minerals of economic interest from mine waste represents a critical strategy for diversifying raw material supply and promoting the goals of a circular economy in mining. However, the reprocessing of these waste materials also introduces environmental impacts. Studying and characterizing these waste dumps, especially in remote and environmentally sensitive areas, is often a time-consuming and challenging effort. Field campaigns required to obtain ground observations involve long-distance travel to sites, resulting in unavoidable disturbance to local ecosystems. In this context, decision making must consider more than economic feasibility alone [173]. Environmental benefits should be weighed against the hidden costs associated with additional energy consumption, emissions, and ecological disruption. These challenges also call for the development of recovery methods that maximize efficiency while minimizing environmental impact. It is crucial to ensure that the reprocessing of mine waste truly reduces the overall environmental burden, rather than simply relocating the waste from one site to another.

#### 4.4.4. Responsibility

The environmental and social challenges brought by mine waste need a collaborative framework involving mining companies, governments, and research institutions, as each stakeholder faces unique limitations. Mining companies usually have the technical expertise but lack regulatory authority; governments have policy tools and decision-making power but primarily face technological limitations; research institutions provide scientific insight but often have limited direct participation in both decision making and exploration. To bridge this gap, clearly defined roles of stakeholders and corporations are preferred. Mining companies play a key role in ensuring transparency by disclosing waste-related information; governments should strengthen regulatory frameworks and enhance public engagement; research institutions can contribute by developing feasible methodologies for waste studies to minimize environmental problems and maximize resource efficiency. The goal is to foster collaboration rather than assign blame. Achieving mining sustainability requires open-minded and collective solutions.

## 5. Conclusions

Waste generated by mining activities remains a major source of global waste streams. These solid mine wastes occupy natural land and can pose serious environmental, safety, and socio-economic risks to ecosystems and communities if not managed properly. Despite these challenges, mine waste may still contain valuable industrial metals. These characteristics further highlight the dual necessity of responsible management and resource recycling.

This review provides an overview of recent advancements in the management and monitoring of mine waste, focusing on sensor-based technologies and data-driven methods for characterization and modeling. A total of 150 journal articles published between 2021 and 2024 were systematically analyzed, with earlier and most recent articles incorporated selectively for background and discussion. The findings highlight recent innovations, a growing trend toward technique integration, and an overall evolution in research focus from characterization to prediction and monitoring.

Each reviewed technique brings unique advantages, challenges and inherent limitations. Ground-based sensors provide highly accurate characterization of the physical, chemical, and mineralogical properties. However, their field applications remain limited due to environmental variabilities and operational regulations. Remote sensing provides powerful spatial–temporal coverage, and airborne platforms are increasingly being explored for high-resolution, cost-effective site monitoring.

Geospatial methods transform sparse field sampling into multi-dimensional models. When combined with drill hole or auger sampling, these approaches can extend to 4D analysis that incorporates temporal variability. This capability supports hazard communication, early warning systems, and spatial–temporal analysis. Yet their application in mine waste studies remains relatively limited. AI-based methods, while their deployment in mine waste studies is still in its infancy, have shown promising performance across various tasks. However, the complexity of waste materials and mathematical calculations remains a challenge.

Several future research priorities emerge from the identified challenges and limitations. Developing accurate and transferable models is a pressing need that requires cross-disciplinary expertise. Considering the technical, regulatory and economic barriers, such a framework could improve the broader adoption of advanced techniques. Advancing real-time, in situ sensor systems helps address the lack of dedicated long-term monitoring methods.

While offering significant advantages, the adoption of cutting-edge techniques also raises critical considerations on environmental and social concerns. Researchers must carefully consider these issues and ensure their research aligns with both local and global regulations governing mining and technological applications. In summary, the management and monitoring of mine waste remain challenging due to the complex nature of waste materials and the technical limitations of existing approaches. Further efforts from both industry and academia will be important to fully unlock the potential of these techniques and support the long-term sustainability of mining activities.

## Figures and Tables

**Table 1:** Example of ground-based sensor technologies and their potential applications.

Sensor Category	Sensor/Methods	Quantitative/Qualitative	Measurement	Application Examples
Geochemical	Inductively coupled plasma mass spectrometry (ICP-MS)	Quantitative	Major, minor, and trace elements	Heavy metal contamination
	X-ray fluorescence (XRF)	Semi-quantitative, quantitative (with proper calibration)	Elemental composition; oxides	Rare earth elements (REEs)
	Laser-induced breakdown spectroscopy (LIBS)	Qualitative/Semi-quantitative	Elemental detection/composition	PTE
	Scanning electron microscopy with energy dispersive spectroscopy (SEM-EDS)	Semi-quantitative/ Quantitative (with proper calibration)	Elemental analysis; mineral structure, textures, and phases	Geochemical; mineral mapping (indirectly)
Mineralogical	Fourier transform infrared spectroscopy (FTIR)	Qualitative/Semi-quantitative (with proper calibration)	Chemical structure; functional groups	Secondary minerals (AMD generation); detect moisture content
	Thermal infrared spectroscopy (TIR)	Qualitative	Thermal emission spectrum; chemical bond	Secondary minerals (AMD generation)
	X-ray diffraction (XRD)	Quantitative	Mineral composition; crystal structure and phase identification; crystallinity	Mineral phases (recoverability), AMD formation potential
	Visible–near infrared (VNIR) spectroscopy	Qualitative/Semi-quantitative (with proper calibration)	Mineral identification; compositional variation	Mapping variability
	Hyperspectral imaging (HSI)	Quantitative/Semi-Quantitative	Mineral mapping; compositional variations	Mapping variability; identifying acid-generating zones
Physical	Thermogravimetric analysis (TGA)	Quantitative	Mass loss; thermal decomposition	Mineral transformation, oxidation, and weathering
	Laser diffraction	Quantitative	Grain size; particle distribution	Difficulty degree in recovering

**Table 2:** Overview of included studies in the ground-based sensor category.

Primary Purpose	Focus	Studies
Environmental Monitoring	Acid/Neutral Mine Drainage	Elghali et al., 2021 [19]
		Çelebi and Ribeiro, 2023 [20]
		Jeong et al., 2021 [21]
		Sapkota et al., 2023 [22]
		Ba et al., 2024 [23]
	Radiological Risks	Moshupya et al., 2022 [24]
	Metal(loid) Contamination	Wen et al., 2022 [25]
		Gürtekin and Aydar, 2023 [26]
		Álvarez-Ayuso et al., 2022 [27]
		Kazamel et al., 2023 [28]
		Mdachi et al., 2024 [29]
	Biogeochemical Monitoring	Courchesne et al., 2021 [13]
	Remediation	Lum et al., 2023 [30]
		Silva de Sá et al., 2023 [31]
		You et al., 2024 [32]
Environmental Monitoring/Secondary Resource Recovery	Metal(loid) Contamination/Base, Precious, Critical Elements	Huljek et al., 2021 [33]
		Islam et al., 2021 [34]
		White et al., 2022 [35]
		Hu et al., 2024 [36]
		Paçarizi et al., 2024 [37]
		Surrette et al., 2024 [38]
		Youpoungam et al., 2024 [39]
Secondary Resource Recovery	Base Metal	Zhang et al., 2021 [40]
		Bevandić et al., 2022 [41]
	Precious Metals	Marín-García et al., 2022 [42]
	REEs	Gómez-Arias et al., 2022 [43]
		Sarker et al., 2023 [44]
		Oliveira et al., 2023 [45]
		Syafrizal et al., 2024 [46]
		Sedda et al., 2024 [47]
	Multiple Types of Elements	Duczmal-Czernikiewicz et al., 2021 [48]
		Sarker et al., 2023 [49]
		Dzinomwa et al., 2023 [50]
		Rosario-Beltré et al., 2023 [51]
		Radu et al., 2024 [52]
		Lohmeier et al., 2024 [53]
Valorization	Element Recovery Pathway	Safhi et al., 2022 [54]
		Trifunović et al., 2024 [3]
		Wang et al., 2024 [55]
	Base Metals	da Silva et al., 2022 [56]
	Upcycling	Amar et al., 2023 [1]
		Mdachi et al., 2024 [57]
	Recovery Pathway and Upcycling	Ferreira et al., 2021 [58]
		Behera and Sahu, 2023 [59]
		Bessa et al., 2024 [60]
		García et al., 2024 [61]
		Gulicovski et al., 2024 [62]
Methodological Innovation	Sensor Data Fusion	Bao et al., 2022 [63]
		Kamps et al., 2024 [64]
	Sampling Designing	Pencea et al., 2023 [65]

**Table 3:** Overview of the included studies using satellite technologies.

Research Purpose	Parameter	Site Location	Use Case	Studies
Safety monitoring	Coal fire	Brazil	Iron ore tailings	Batoni et al., 2024 [67]
		Poland	Coal mine heap and tailings	Worsa-Kozak et al., 2024 [68]
	Stability	Spain	Mining area (including waste)	López-Vinielles et al., 2021 [69]
		Brazil	Iron ore tailings dam	Grebby et al., 2021 [70]
		Myanmar	Jade-mine-related tailings	Lin et al., 2021 [71]
		China	Mine waste dump	Tabish et al., 2022 [72]
		China	Tailings area	Tan et al., 2022 [73]
		China	Tailings reservoir	Wu et al., 2022 [74]
		Brazil	Iron-mining-related waste	Gama et al., 2022 [75]
		South Korea	Limestone mine and TSF	Lee et al., 2022 [76]
		Peru; Chile; South Africa; Tanzania	TSF water pond	Cacciuttolo and Cano, 2023 [77]
		China	Copper mine tailings pond	Xie et al., 2023 [78]
		China	Mineral waste dump	Duan et al., 2023 [79]
		South Africa	Diamond mine waste	Torres-Cruz and O’Donovan, 2023 [80]
		Spain; Brazil; Australia	Failure tailings	Mirmazloumi et al., 2023 [81]
		Brazil	Iron ore mine tailings	Das et al., 2024 [82]
		China	Iron ore tailings pond	Ding et al., 2024 [83]
		China; Brazil	Tailings dam	Yan et al., 2024 [84]
		Australia	Tailings dam	Bayaraa et al., 2024 [85]
		China; India; Mexico; Australia; Brazil	Failure tailings	Rana et al., 2024 [86]
Environmental monitoring	Stability	China	Tailings and active mine	Du et al., 2021 [87]
	PTE	Mexico	Tailings in town	Ahumada-Mexía et al., 2021 [6]
	Heavy metal	Tunisia	Pb-Zn tailings and soil	Mezned et al., 2022 [88]
	Dust dispersion	Mongolia	mine dump	Batbold et al., 2022 [89]
	Remediation	Argentina	Historical uranium site	Castaño et al., 2023 [90]
	Localization	USA	Uranium mine waste feature	Hubbard et al., 2023 [91]
		Russia	Mine waste	Galchenko et al., 2024 [92]
	AMD Potential	Turkey	Coal mine waste	Soydan et al., 2021 [93]
		Iran	Copper mine waste	Hosseinjanizadeh et al., 2023 [94]
Secondary resource	Precious and critical element recovery	India	Fe-Mn-rich residues	Guglietta et al., 2022 [95]
		South Africa	Gold tailings	Zhang et al., 2023 [7]
		Indonesia	Bauxite mining area	Nugraheni et al., 2023 [96]

**Table 4:** Overview of the included studies using airborne sensors.

Research Purpose	Parameters	Site Location	Use Case	Sensor Setting	Studies
Safety monitoring	Coal fire	Poland	Coal waste dump	Thermal imaging	Różański et al., 2022 [97]
		Czech Republic	Coal mine waste	Infrared camera	Zubíček et al., 2024 [98]
		China	Coal gangue	Visible and thermal camera	Ma et al., 2024 [99]
	Stability	Brazil	Bauxite tailings	Imaging camera	Lima Filho et al., 2023 [100]
		Australia	Coal mine dump	Optical camera	Thiruchittampalam et al., 2024 [101]
		Caribbean Archipelago	Waste dump	RGB camera	Arrieta and Zhang, 2024 [102]
		China	Tailings	Five-angle camera	Zhi et al., 2023 [103]
		China	Tailings pond	Five-lens tilt camera	Zhang et al., 2023 [104]
		China	Tailings pond	Not specified	Wang et al., 2024 [105]
Environmental monitoring	AMD potential	USA	Waste rock pile	VNIR sensors	Cramer et al., 2021 [106]
	Radiology risks	Kyrgyzstan; Kazakhstan	Uranium waste site	Gamma spectroscopy	Kunze et al., 2022 [107]
	UAV positioning accuracy	South Korea	Mine waste site	Not specified	Kim et al., 2023 [108]
Secondary resource	Economic potential	Portugal	Mine dump	RGB camera	de Oliveira et al., 2024 [109]
		Cyprus	Sulfide mine waste	Imaging camera	Saratsis et al., 2023 [110]

**Table 5:** Overview of the included studies using a LiDAR device.

Research Purpose	Parameters	Site Location	Use Case	Studies
Environmental Monitoring	Costal impact	USA	Tailings	Kerfoot et al., 2021 [111]
	Erosion	Spain	Tailings pond	Martín-Velázquez et al., 2022 [112]
	Pollutant element	Spain	Tailings	Martín-Crespo et al., 2023 [113]
	Remediation assessment	USA	Uranium mining region waste	Stengel et al., 2024 [114]

**Table 6:** Parameters of satellites that are commonly used by the reviewed articles. Source: [115].

Satellite		Operator	Type	Launch	End of Life	Revisit Interval
Sentinel-	1A	European Space Agency (ESA)	SAR	2014	2022	6 days
	1B			2016	2022	
	2A		Optical	2015	In operation	5 days
	2B			2017		
Landsat-	5	NASA, USGS	Optical, thermal	1984	2013	16 days
	8			2013	In operation	8 days
	9			2021	In operation	
Ziyuan 3		China Ministry of Natural Resources	Optical	2012	2023	5 days
Himawari 8		Japan Meteorological Agency (JMA)	Optical, thermal	2014	In operation	Geostationary orbital
WorldView-3 (Commercial)		DigitalGlobe	Optical	2014	In operation	<1 day
Pléiades (Civil, Commercial)		French National Space Agency	Optical	2011; 2012	In operation	<1 day

**Table 7:** Comparative evaluation of ground-based sensors and remote sensing technologies.

Factors	Ground-Based Sensors	Satellite/Airborne Remote Sensing
Spatial coverage	Low/Localized	High/Regional–Global
Spatial resolution	High	Moderate–High
Temporal capability	Low–High	Moderate
Real-time performance	Possible	Limited
Accuracy	High	Moderate
Operational cost	Moderate–High	Low–Moderate
Computational demand	Moderate	High
Scalability	Low–Moderate	High
Key strengths	High accuracy, rapid measurement, in situ validation, continuous monitoring are possible	Large coverage, spatial continuity, enables monitoring of areas with limited physical access
Key limitations	Limited spatial representativeness	Complex calculation and processing, lower local accuracy
Typical applications	Characterization, validation	Mapping and monitoring

**Table 9:** Overview of reviewed studies using AI-based methods.

Application	Use Cases	Site Location	Main Models Used	Citation
Detection	Tailings ponds	China	YOLOv4; RF	Lyu et al., 2021 [137]
	Tailings ponds	China	Faster R-CNN	Yan et al., 2021 [138]
	Tailings ponds	China	Faster R-CNN	Yan et al., 2022 [139]
	Tailings ponds	China	Improved YOLOv5s	Sun et al., 2023 [140]
	Waste rock dumps; leaching waste dumps	Chile	Vision Transformer (ViT) Classifier; YOLOv7; Mask R-CNN	Silva et al., 2023 [141]
	Tailings ponds	China	YOLOv7	Ge et al., 2024 [142]
	Tailings ponds	China	YOLOv5	Zheng et al., 2024 [143]
Segmentation	Tailings ponds	China	Scene-Classification-Sematic-Segmentation model	Wang et al., 2023 [144]
	Tailings ponds	China	Pseudo-Siamese Visual Geometry Group Encoder–Decoder network	Zhang et al., 2023 [145]
	Tailings dams	/	CNN; U-Net	Gomez et al., 2024 [146]
	Tailings ponds	China	YOLOv5-seg; Canny Edge Detection	Duan et al., 2024 [147]
Classification	Tailings dams	Chile	RF, SVM, ANN, XGBoost	Pacheco et al., 2022 [148]
	Tailings pond	China; Australia; Malaysia	Deep Neural Network (DDN); ResNet-50	Yu and Zahidi, 2023 [149]
	Tailings	Finland	ANN	Herrera et al., 2024 [150]
Regression	Tin–polymetallic mining area	China	PLSR; Back Propagation Neural Networks (BPNN)	Bian et al., 2021 [151]
	TSFs	South Africa	K-Nearest Neighbors (kNN); RF; AdaBoost	Zhang et al., 2023 [152]
	Mine tailings, soil, sediments	China	PLSR; Extreme Learning Machine (ELM)	Bian et al., 2022 [153]
	Waste dump of Cu mine	Spain	NN	Expósito-Suárez et al., 2024 [154]
	Tailings	Germany; Portugal	GBRT	Zheng et al., 2023 [155]
	Tailings and mine waste	New Zealand; USA	RF with Bayesian Optimization	Qi et al., 2024 [156]
	Metal mine tailings ponds	China	Bidirectional Long and Short Memory Network Model	Jing and Gao, 2022 [157]
	TSFs	Australia	EE-DL	Bayaraa et al., 2023 [158]
Regression and classification	Tailings	China	Autoencoder, K-Means	Xiao et al., 2022 [159]
	Tailings	Mongolia	PLSR; RF; kNN, LR, SVM, NN	Ogen et al., 2022 [160]
	Thickened tailings	Chile	ANN; SVM; RF	Arancibia et al., 2021 [161]
Novel framework	Gold mine tailings reservoirs	China	Brain-Inspired Visual Cortex-Based Data-Driven Automatic ML Interpretation Method; Deep Full CNN	Wan et al., 2023 [162]
	Tailings pond	China	NN—Temporal Convolutional Network	Nie et al., 2024 [163]

**Table 10:** Overview of reviewed studies in the Geodatabase category.

Author	Studied Location	Scale	Method	Focus
Sarker et al., 2022 [164]	Australia	National	Visual inspection, public data integration	Location; name; commodities; operational statues
Gomez et al., 2022 [165]	Australia, Chile, Mexico	Multi-national	Public data integration	Critical elements
Mpanza, 2024 [166]	Gauteng	Province	Public data visualization	Location; operational statuses
Massignan and Sánchez, 2024 [167]	World	Global	Database review	Existing database

## Data Availability

No new data were created or analyzed in this study.
